# Author Correction: An extrinsic motor directs chromatin loop formation by cohesin

**DOI:** 10.1038/s44318-024-00341-9

**Published:** 2025-01-31

**Authors:** Thomas M Guérin, Christopher Barrington, Georgii Pobegalov, Maxim I Molodtsov, Frank Uhlmann

**Affiliations:** 1 Chromosome Segregation Laboratory, The Francis Crick Institute, London, UK; 2 Université Paris Cité and Université Paris-Saclay, Inserm, CEA, Stabilité Génétique Cellules Souches et Radiations, Fontenay-aux-Roses, France; 3 Bioinformatics & Biostatistics Science Technology Platform, The Francis Crick Institute, London, UK; 4 Mechanobiology and Biophysics Laboratory, The Francis Crick Institute, London, UK; 5 Department of Physics and Astronomy, University College London, London, UK

## Abstract

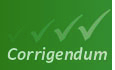

**Correction to:** *The EMBO Journal* (2024) 43: 4173–4196. 10.1038/s44318-024-00202-5 | Published online 19 August 2024

The authors contacted the journal after identifying that the figure panels in Figs. 2D and EV4C were not generated using the indicated yeast strain. The authors have provided the journal with the correct data. After reviewing the corrected data, the journal withdraws and replaces the following figures.

**Figure 2D is withdrawn and replaced**

**Figure 2D:**
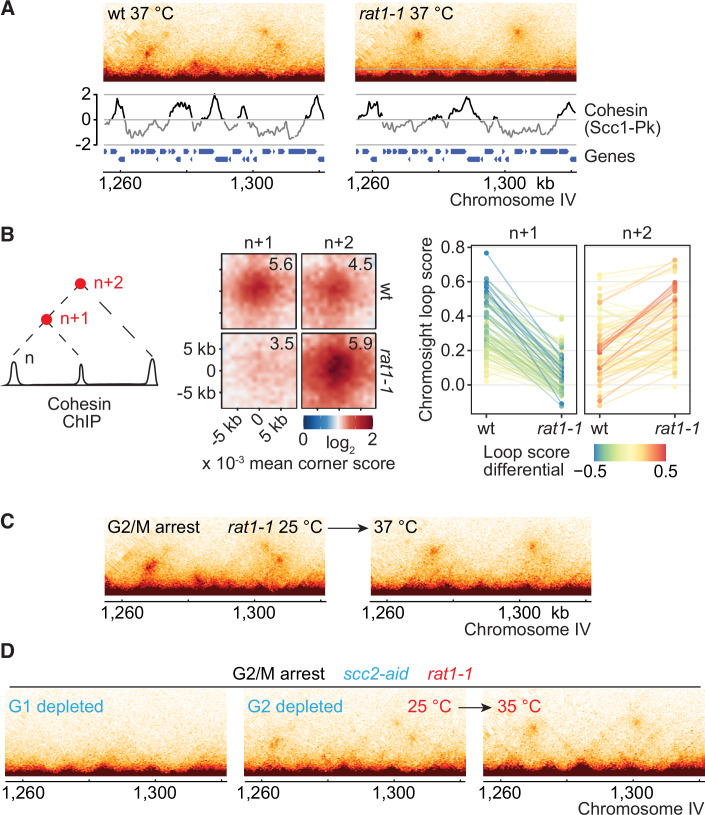
Corrected.

**Figure 2D:**
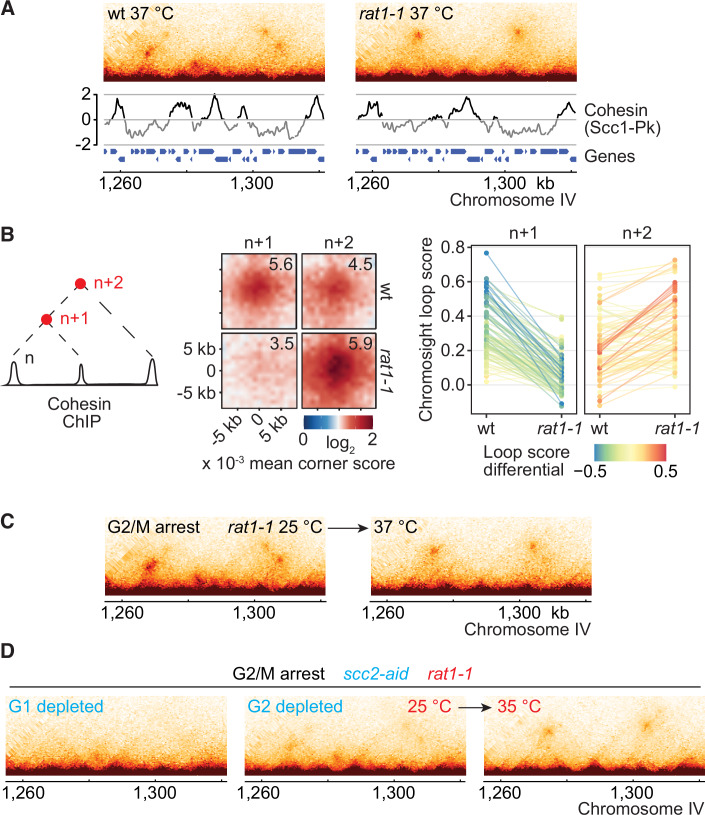
Original.

**Figure EV4C is withdrawn and replaced**

**Figure 4C:**
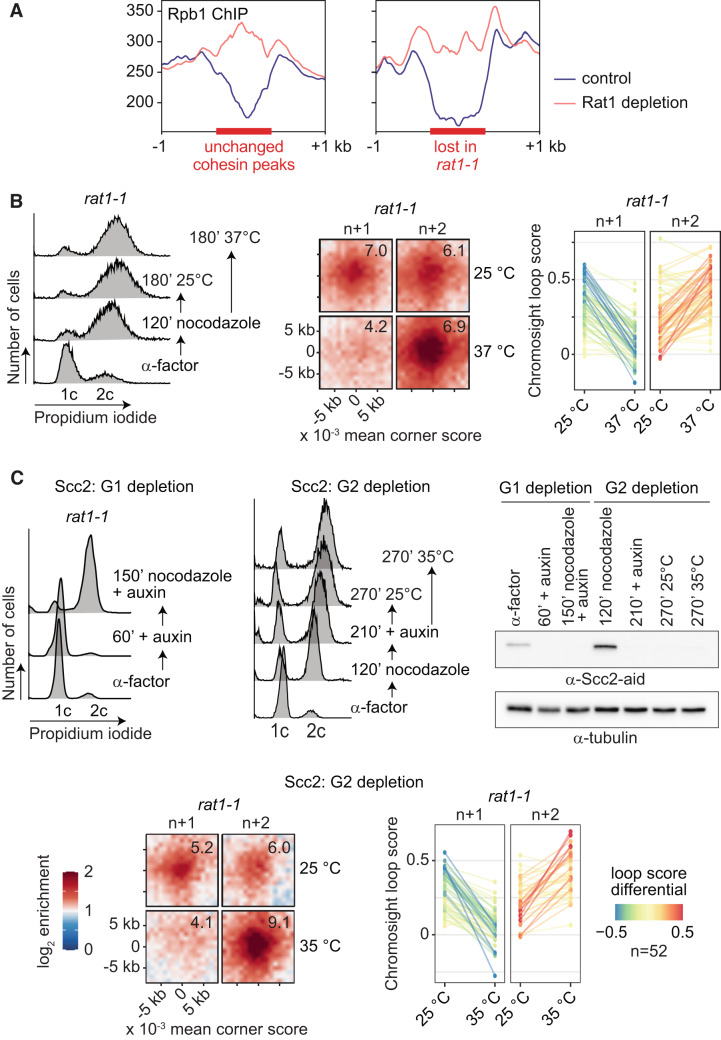
Corrected.

**Figure EV4C legend is corrected**

**Figure 4C:**
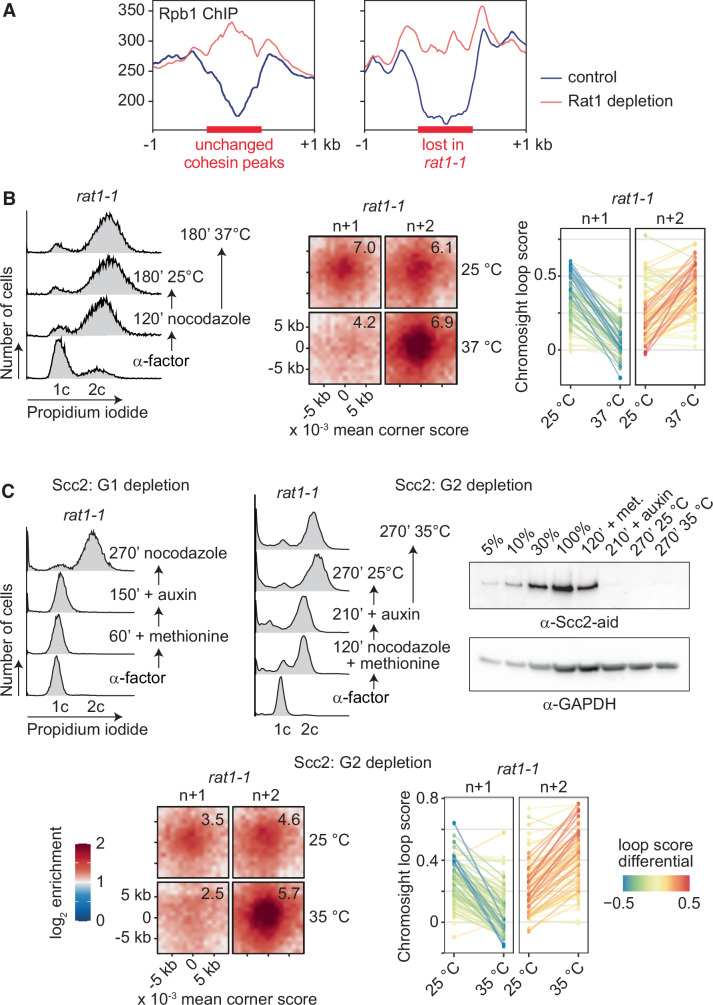
Original.

Source files for figure panel EV4C**are published with this correction**.

The Fig. EV4C legend is corrected to (changes in bold):

(**C**) FACS analyses of DNA content of the cells in the experiment shown in Fig. 2D, together with experimental outlines. Western blot analysis confirmed Scc2 depletion **by an auxin-inducible degron. Samples at the indicated times in the experiment are shown**. Scc2 was detected using the aid-tag antibody. **Tubulin** served as a loading control. Aggregate loop profiles (*n* = **52**) and a graph depicting the *rat1-1* dependent loop score changes are shown.

Author statement:

Figures 2D and EV4C in Guérin et al. 2024 were meant to document that the cohesin loader is required for cohesin-dependent chromatin loop formation, but not for the growth of these loops in response to transcriptional readthrough (following Rat1 inactivation). We have become aware that the yeast strain used for the presented experiment was in fact (Y6859: *Mat***a** *URA3::pMET3-sth1-aid::KAN*^*MX6*^ *pADH1-OsTIR1-myc*_*9*_*::ADE2 SMC3-PK*_*3*_*::HIS3*^*MX6*^ *rat1-1::LEU2*) carrying a degron tag on the RSC chromatin remodeller large subunit Sth1. The experiment therefore shows that the RSC chromatin remodeller is required for chromatin loop formation, but not for their enlargement by pervasive transcription. The experiment using the intended strain (Y7013*: Mat***a** *pADH1-OsTIR1-myc*_*9*_*::ADE2 scc2-IAA17::KAN*^*MX6*^ *rat1-1::LEU2*) to deplete the cohesin loader, is shown below. The outcomes observed following Scc2 depletion, or following Sth1 depletion, are qualitatively indistinguishable, and all our conclusions remain unchanged. We have updated the GEO micro-C data submission accordingly, and we apologise for any inconvenience caused by this correction.

All authors agree to this author correction.

## Supplementary information

Source Data EV4C_Corrected

