# Supplementary material for: An extrinsic motor directs chromatin loop formation by cohesin
Source: EMBO J. 2024 Aug 19;43(19):3. doi: 10.1038/s44318-024-00202-5 (PMC11445435; doi:10.1038/s44318-024-00202-5)
Supplement: Supplementary file 1 — Appendix [file 44318_2024_202_MOESM1_ESM.docx]

**An extrinsic motor directs cohesin chromatin loop formation and expansion**

Thomas M Guérin, Christopher Barrington, Georgii Pobegalov, Maxim I Molodtsov &

Frank Uhlmann

**Appendix**

**Table of content:**

**Appendix Table S1. Yeast strains used in this study. 2**

**Appendix Table S2. micro-C library sequencing read count statistics. 4**

**Appendix Figure S1. Supporting analyses: evidence for unwound DNA as possible cohesin target for loop capture. 7**

**Appendix Figure S2. Supporting analyses: TAD formation without cohesin. 8**

**Appendix Figure S3. Reproducibility between micro-C experiments. 9**

**Appendix Table S1 – Yeast strains used in this study.**

All strains were derivative of the W303 background (strain *141*), unless otherwise stated.

| **Figure 1:** |  |
| --- | --- |
| **141** | *MAT***a** *leu2-3,112 trp1-1 can1-100 ura3-1 ade2-1 his3-115* |
| **5226** | *MAT***a** *can1-100, pep4∆::HIS3 wpl1∆::LEU2 eco1∆::KAN^MX6^ pRSII402-pGAL1/10-GAL4+SMC1-Pk_3_::ADE2* YIplac204*-pGAL1/10-SMC3+SCC1-3C-2PROTA::TRP1* YIplac211*-pGAL1/10-SCC3-MYC1::URA3* |
| **6853** | *MAT***a** *can1-100 pep4∆::HIS3 wpl1∆::LEU2 eco1∆::KAN^MX6^ SCC3^K423E, K520E, K669E^-myc::NAT^R^ pRSII402-pGAL1/10-GAL4+SMC1-Pk_3_::ADE2* YIplac204*-pGAL1/10-SMC3+SCC1-3C-2PROTA::TRP1* YIplac211*-pGAL-SCC3^K423E, K520E, K669E^-myc::URA3* |
| **6854** | *MAT***a** *can1-100, pep4∆::HIS3 wpl1∆::LEU2 eco1∆::KAN^MX6^ SMC1^R53E, R58E, N60E, K63E^-Pk_3_::NAT^R^ pRSII402-pGAL1/10-GAL4+SMC1^R53E, R58E, N60E, K63E^-Pk_3_::ADE2* YIplac204*-pGAL1/10-SMC3+SCC1-3C-2PROTA::TRP1* YIplac211*-pGAL1/10-SCC3-MYC1::URA3* |
| **6918** | *MAT***a** *SCC3-myc::URA3* |
| **6920** | *MAT***a** *SCC3^K423E,K510E,K669E^-myc::URA3* |
| **5278** | *MAT***a** *can1-100 pep4Δ::HIS3 pGAL1/10-SCC2-3C-2PROTA::ADE2 pGAL1/10-SCC4-HA3::TRP1* |
| **6855** | *MAT***a** *SCC3-myc::URA3 SMC3-Pk_3_::HPH^R^* |
| **6856** | *MAT***a** *SCC3^K423E, K520E, K669E^-myc::URA3 SMC3-Pk_3_::HPH^R^* |
| **6857** | *MAT***a** *SMC1-Pk_3_::ADE2* |
| **6858** | *MAT****a*** *tor1-1 fpr1∆::NAT^R^ SMC3-Pk_3_::HPH^R^ pGAL1/10-SIC1^V5,V33 A76^-HA::ADE2 RPL13A-2xFKBP12::TRP1 rpb1-GFP-FRB::HIS3^MX^ rpb3-FRB::HIS3^MX^* |
| **6795** | *MAT***a** *SMC1^R53E, R58E, N60E, K63E^-Pk_3_::ADE2* |
| **6862** | *Candida glabrata,* (NCYC 388 background) *CAGL0H02805g-Pk_3_::HPH^R^* |

***Figure 2:***

| **2271** | FY23 background MAT**a** leu2Δ1 ura3−52 trp1Δ63 SCC1-Pk_9_::TRP1 |
| --- | --- |
| **2272** | FY23 background *MAT***a** *rat1-1 leu2Δ1 ura3−52 trp1Δ63 SCC1-Pk_9_::TRP1* |
| **6859** | *MAT***a** *rat1-1::LEU2 URA3::pMET3-scc2-aid::KAN^MX^ pADH1-OsTIR1-myc_9_::ADE2 SMC3-Pk_3_::HIS3^MX^* |

***Figure 3:***

| **6858** | As above, Figure 1 |
| --- | --- |
| **6312** | *MAT***a** *tor1-1 fpr1∆::NAT^R^ SMC3-Pk_3_::HPH^R^ pGAL1/10-SIC1^V5,V33 A76^-HA::URA3 RPL13A-2xFKBP12::TRP1 rpb3-FRB::HIS3^MX^* |
| **6236** | *MAT***a** *tor1-1 fpr1∆::NAT^R^ SMC3-Pk_3_::HPH^R^ pGAL1/10-SIC1^V5 V33,A76^-HA::URA3 RPL13A-2xFKBP12::TRP1 rpb1-GFP-FRB::HIS3MX* |
| **6217** | *MAT***a** *scc1-aid::KAN^R^ pGPD1-OsTIR1::LEU2 SMC3-Pk_3_::HIS3^MX^* |

***Figure 4:***

| **6900** | MAT**a/a** top2-4 top1∆::KlTRP1 |
| --- | --- |
| **6901** | *MAT***a/a** *top2-4 top1∆::KlTRP1 pGPD-TopA::LEU2* |
| **6863** | *MAT***a** *sub1Δ::KlTRP1* |
| **6750** | *MAT****a*** *pGAL1-10-SIC1^V5,V33 A76^::TRP1* |
| **6875** | *MAT***a** *pGAL1-10- SIC1^V5,V33 A76^::ADE2 chl1Δ::KlTRP1* |

***Figure 5:***

| **6217, 6858** | As above, Figure 3 |
| --- | --- |

***Figure EV2:***

| **193** | MAT**a** ura3::3XURA3-tetO112 his3::HIS3-tetR-GFP |
| --- | --- |
| **6825** | *MAT***a** *ura3::3XURA3-tetO112 his3::HIS3-tetR-GFP SCC3^K423E, K520E, K669E^-myc::NAT^R^* |
| **6860** | *MAT***a** *ura3::3XURA3-tetO112 his3::HIS3-tetR-GFP SMC1^R53E, R58E, N60E, K63E^-Pk_3_::ADE2* |
| **4169** | *MAT***a** *ura3::3XURA3-tetO112 his3::HIS3-tetR-GFP chl1Δ::LEU2* |
| **2316** | *MAT***a** *rad52Δ::LEU2* |
| **6929** | *MAT***a/a** *SMC1-Pk_3_::ADE2/ SMC1-Pk_3_::ADE2* |
| **6928** | *MAT***a/a** *SCC3^K423E, K520E, K669E^-myc::URA3/ SCC3^K423E, K520E, K669E^-myc::URA3* |
| **6930** | *MAT***a/a** *SMC1^R53E, R58E, N60E, K63E^-Pk_3_::ADE2/ SMC1^R53E, R58E, N60E, K63E^-Pk_3_::ADE2* |

***Figure EV3:***

| **6885** | MAT**a** pADH1-OsTIR1-myc_9_::ADE2 scc3-aid ::KlLEU2 |
| --- | --- |
| **6891** | *MAT***a** *pADH1-OsTIR1-myc_9_::ADE2 HIS3::pMET3 -scc3-aid ::KlLEU2* |

**Appendix Table S2. micro-C library sequencing read count statistics.**

|  | **dataset** | **nofilter** | **mapq30** | **mapq30_cis150** |
| --- | --- | --- | --- | --- |
| **Figure 1** |  |  |  |  |
| control_G2 * | control_G2 * | 120,947,404 | 118,680,029 | 114,505,836 |
| GUE3552A29 | control_G2_N1 | 34,516,587 | 33,835,088 | 32,417,515 |
| GUE3552A31 | control_G2_N2 | 86,430,817 | 84,844,941 | 82,088,321 |
| Scc3-3E * | Scc3-Patch3 * | 108,528,282 | 106,414,468 | 102,279,975 |
| GUE3552A75 | Scc3-Patch3_N1 | 56,227,606 | 55,142,240 | 53,023,039 |
| GUE3552A76 | Scc3-Patch3_N2 | 52,300,676 | 51,272,228 | 49,256,936 |
| Smc1-4E * | Smc1-4E * | 102,038,149 | 100,025,185 | 95,766,673 |
| GUE3552A77 | Smc1-4E_N1 | 51,981,280 | 50,951,338 | 48,841,365 |
| GUE3552A78 | Smc1-4E_N2 | 50,056,869 | 49,073,847 | 46,925,308 |
|  |  |  |  |  |
| **Figure 2** |  |  |  |  |
| wt_37C_G2 * | wt_37C_G2 * | 75,510,911 | 74,000,781 | 71,112,317 |
| GUE3552A18 | wt_37C_G2_N1 | 42,849,578 | 42,028,642 | 40,485,936 |
| GUE3552A34 | wt_37C_G2_N2 | 32,661,333 | 31,972,139 | 30,626,381 |
| rat1_37C_G2 * | rat1_37C_G2 * | 102,335,992 | 100,285,709 | 96,636,249 |
| GUE3552A19 | rat1_37C_G2_N1 | 66,849,150 | 65,523,313 | 63,235,547 |
| GUE3552A33 | rat1_37C_G2_N2 | 35,486,842 | 34,762,396 | 33,400,702 |
| rat1_25C_G2 * | 2272_HS * | 73,253,286 | 71,859,782 | 68,823,043 |
| GUE3552A53 | 2272_HS_N1 | 32,995,252 | 32,354,899 | 30,967,951 |
| GUE3552A58 | 2272_HS_N2 | 40,258,034 | 39,504,883 | 37,855,092 |
| rat1_37C_G2 * | 2272_No_HS * | 94,162,912 | 92,483,299 | 89,012,298 |
| GUE3552A52 | 2272_No_HS_N1 | 52,074,757 | 51,138,752 | 49,296,089 |
| GUE3552A57 | 2272_No_HS_N2 | 42,088,155 | 41,344,547 | 39,716,209 |
| rat1_depleted_G1 * | TG75_Scc2_G1Depl * | 76,457,158 | 75,058,764 | 71,718,303 |
| GUE3552A51 | TG75_Scc2_G1Depl_N1 | 37,642,151 | 36,934,138 | 35,282,496 |
| GUE3552A56 | TG75_Scc2_G1Depl_N2 | 38,815,007 | 38,124,626 | 36,435,807 |
| rat1_depleted_G2_25 * | TG75_Scc2_G2Depl_25 * | 132,185,432 | 129,665,114 | 124,035,198 |
| GUE3552A97 | TG75_Scc2_G2Depl_25_N1 | 74,171,507 | 72,773,493 | 69,630,595 |
| GUE3552A99 | TG75_Scc2_G2Depl_25_N2 | 58,013,925 | 56,891,621 | 54,404,603 |
| rat1_depleted_G2_35 * | TG75_Scc2_G2Depl_35 * | 135,307,182 | 132,877,535 | 125,986,105 |
| GUE3552A98 | TG75_Scc2_G2Depl_35_N1 | 69,617,289 | 68,382,279 | 64,882,462 |
| GUE3552A100 | TG75_Scc2_G2Depl_35_N2 | 65,689,893 | 64,495,256 | 61,103,643 |

|  |  |  |  |  |
| --- | --- | --- | --- | --- |
|  | **dataset** | **nofilter** | **mapq30** | **mapq30_cis150** |
| **Figure 3** |  |  |  |  |
| rapamycin_G2 * | rapamycin_G2 * | 58,140,938 | 56,827,439 | 54,321,534 |
| GUE3552A30 | rapamycin_G2_N1 | 29,920,007 | 29,254,395 | 28,026,870 |
| GUE3552A32 | rapamycin_G2_N2 | 28,220,931 | 27,573,044 | 26,294,664 |
| control_G1 * | control_G1 * | 76,143,016 | 74,636,686 | 71,150,706 |
| GUE3552A12 | control_G1_N1 | 38,463,623 | 37,664,098 | 35,968,935 |
| GUE3552A15 | control_G1_N2 | 37,679,393 | 36,972,588 | 35,181,771 |
| rapamycin_G1 * | rapamycin_G1 * | 76,006,404 | 74,215,951 | 70,358,452 |
| GUE3552A13 | rapamycin_G1_N1 | 43,347,065 | 42,376,984 | 40,049,813 |
| GUE3552A16 | rapamycin_G1_N2 | 32,659,339 | 31,838,967 | 30,308,639 |
| 6217_G2_auxin * | 6217_G2_auxin * | 104,678,620 | 102,601,741 | 97,432,051 |
| GUE3552A26 | 6217_G2_auxin_N1 | 65,750,249 | 64,483,372 | 61,253,069 |
| GUE3552A28 | 6217_G2_auxin_N2 | 38,928,371 | 38,118,369 | 36,178,982 |
|  |  |  |  |  |

| **Figure 4** |  |  |  |  |
| --- | --- | --- | --- | --- |
| 6900_top2-4_top1 * | 6900_top2-4_top1 * | 70,094,547 | 68,281,835 | 64,066,203 |
| GUE3552A131 | 6900_top2-4_top1_N1 | 26,775,548 | 26,067,655 | 24,521,252 |
| GUE3552A132 | 6900_top2-4_top1_N2 | 26,287,511 | 25,583,421 | 24,008,772 |
| GUE3552A133 | 6900_top2-4_top1_N3 | 17,031,488 | 16,630,759 | 15,536,179 |
| 6901_top2-4_top1_topA * | 6901_top2-4_top1_topA * | 97,146,176 | 95,015,090 | 92,926,972 |
| GUE3552A134 | 6901_top2-4_top1_topA_N1 | 38,129,431 | 37,343,240 | 36,621,010 |
| GUE3552A135 | 6901_top2-4_top1_topA_N2 | 37,799,253 | 36,990,630 | 36,264,148 |
| GUE3552A136 | 6901_top2-4_top1_topA_N3 | 21,217,492 | 20,681,220 | 20,041,814 |
| 141_WT_chl1sub1 * | 141_WT_chl1sub1 * | 59,034,400 | 57,931,084 | 55,937,050 |
| GUE3552A104 | 141_WT_chl1sub1_N1 | 59,034,400 | 57,931,084 | 55,937,050 |
| 6863_sub1 * | 6863_sub1 * | 68,505,150 | 67,183,067 | 64,721,634 |
| GUE3552A106 | 6863_sub1_N1 | 68,505,150 | 67,183,067 | 64,721,634 |
| 6750_WT_G1 * | 6750_WT_G1 * | 77,155,055 | 75,603,330 | 71,423,271 |
| GUE3552A126 | 6750_WT_G1_N1 | 41,741,388 | 40,863,325 | 38,647,227 |
| GUE3552A137 | 6750_WT_G1_N2 | 35,413,667 | 34,740,005 | 32,776,044 |
| 6875_chl1_G1 * | 6875_chl1_G1 * | 76,620,044 | 75,113,791 | 70,931,136 |
| GUE3552A127 | 6875_chl1_G1_N1 | 39,634,321 | 38,839,985 | 36,674,665 |
| GUE3552A138 | 6875_chl1_G1_N2 | 36,985,723 | 36,273,806 | 34,256,471 |

|  | **dataset** | **nofilter** | **mapq30** | **mapq30_cis150** |
| --- | --- | --- | --- | --- |
| **Figure 5** |  |  |  |  |
| 6217_IAA_Raffinose * | 6217_IAA_Raffinose * | 77,117,929 | 75,344,015 | 70,442,004 |
| GUE3552A93 | 6217_IAA_Raffinose_N1 | 35,502,295 | 34,667,630 | 32,404,750 |
| GUE3552A94 | 6217_IAA_Raffinose_N2 | 41,615,634 | 40,676,385 | 38,037,254 |
| 6217_IAA_Galactose * | 6217_IAA_Galactose * | 78,071,149 | 76,187,755 | 71,368,878 |
| GUE3552A95 | 6217_IAA_Galactose_N1 | 43,147,697 | 42,076,318 | 39,478,535 |
| GUE3552A96 | 6217_IAA_Galactose_N2 | 34,923,452 | 34,111,437 | 31,890,343 |

| **Figure EV3** |  |  |  |  |
| --- | --- | --- | --- | --- |
| 6885_Scc3-AID_Untreated * | 6885_Scc3-AID_Untreated * | 63,464,011 | 62,134,795 | 59,415,256 |
| GUE3552A116 | 6885_Scc3-AID_Untreated_N1 | 29,249,952 | 28,636,560 | 27,334,454 |
| GUE3552A117 | 6885_Scc3-AID_Untreated_N2 | 34,214,059 | 33,498,235 | 32,080,802 |
| 6891_Scc3-AID_Treated * | 6891_Scc3-AID_Treated * | 74,913,795 | 73,381,245 | 69,999,279 |
| GUE3552A118 | 6891_Scc3-AID_Treated_N1 | 34,906,726 | 34,176,181 | 32,581,153 |
| GUE3552A119 | 6891_Scc3-AID_Treated_N2 | 40,007,069 | 39,205,064 | 37,418,126 |


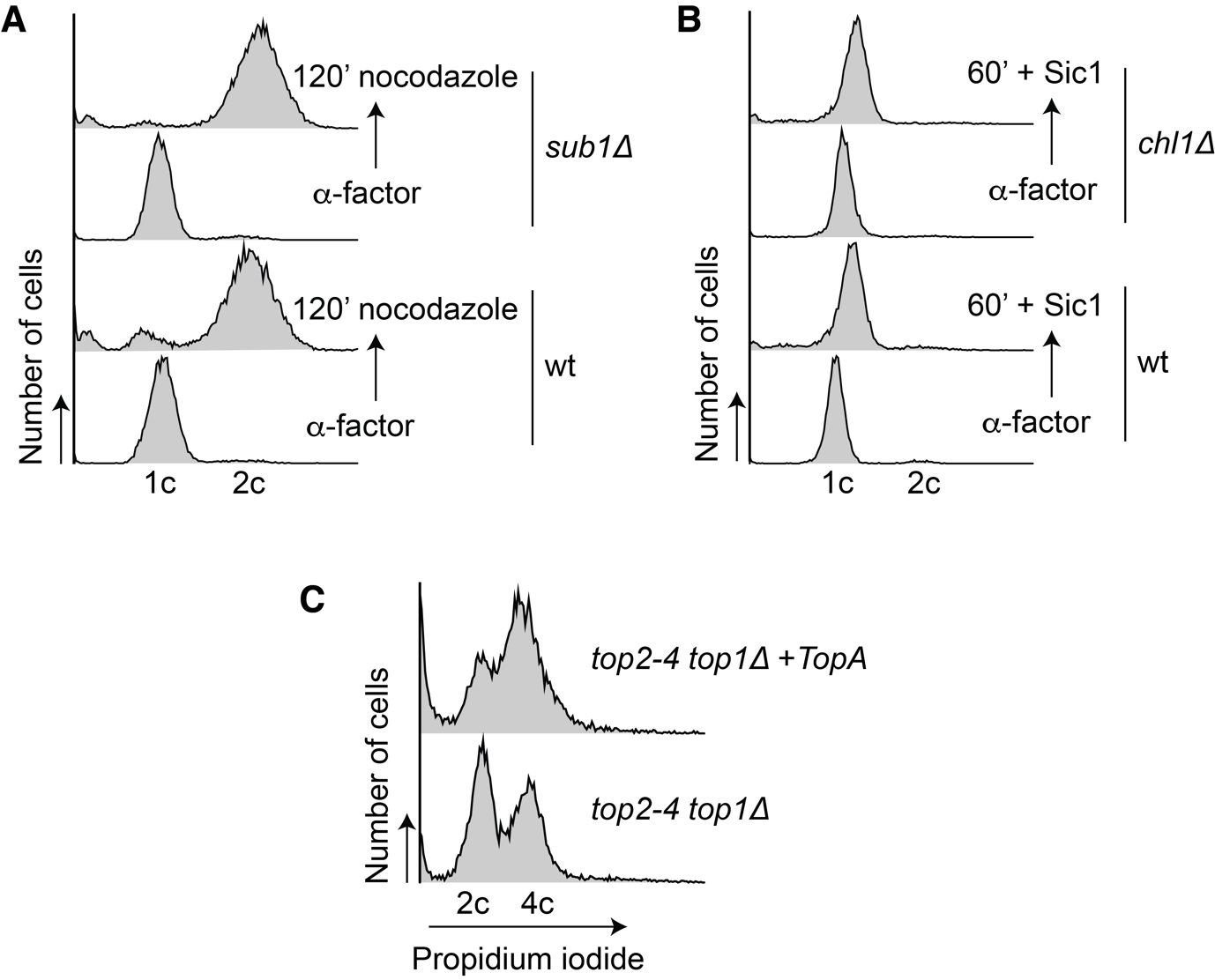


**Appendix Figure S1. Supporting analyses: evidence for unwound DNA as possible cohesin target for loop capture.**

**(A)** - **(C)** FACS analyses of DNA content and experimental outlines of the three experiments shown in Figure 4. (**A**) wild type (wt) and *sub1Δ* cells were synchronised in G1 by α-factor treatment and released into nocodazole containing medium for arrest in G2/M, when micro-C samples were taken. (**B**) wild type and *chl1Δ* cells were released from synchronisation by α-factor treatment into a late G1 arrest due to Sic1 overexpression. The previously reported drop of chromosomal cohesin levels in *chl1Δ* cells during DNA replication (Samora *et al*, 2016), would have confounded a comparison in G2/M. (**C**) We reproduced previously established experimental conditions (Joshi *et al*, 2010), employing asynchronous cultures during exponential growth phase, shifted to 35°C for two hours to achieve *top2-4* inactivation. The FACS analysis revealed a relative shift of *top1Δ top2-4* +*TopA* cells to G2 DNA content during this treatment, a cell cycle phase in which cohesin chromatin loops are expected to be more stable. In contrast to this expectation, *top1Δ top2-4* +*TopA* cells show reduced looping. Note that *top1Δ top2-4* cells grow as diploids.


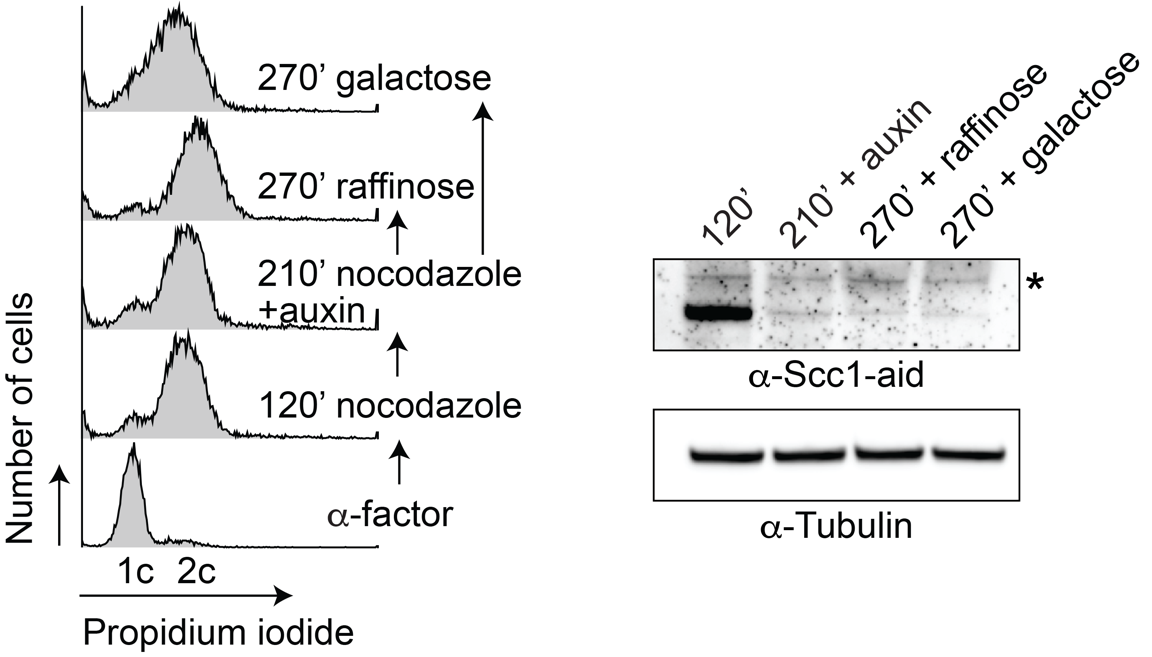


**Appendix Figure S2. Supporting analyses: TAD formation without cohesin.**

FACS analysis of DNA content of the cells in the experiment shown in Fig. 5, as well as an experimental outline. Western blot analysis confirmed Scc1-aid depletion by its auxin-inducible degron, following auxin addition to a G2/M arrested culture. The asterisk marks a background band recognised by the α-aid-tag antibody. Tubulin served as a loading control.

**
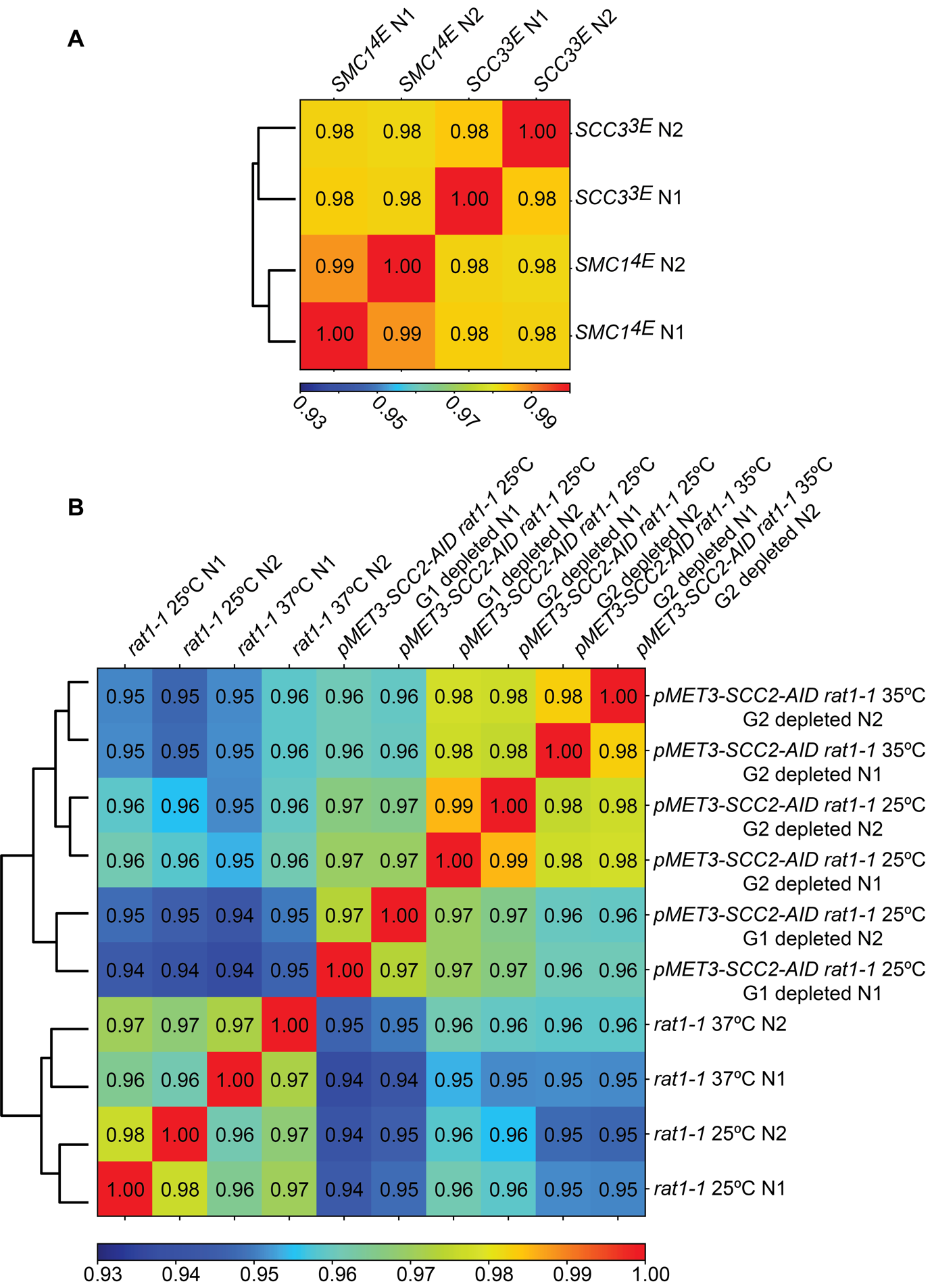
**

**
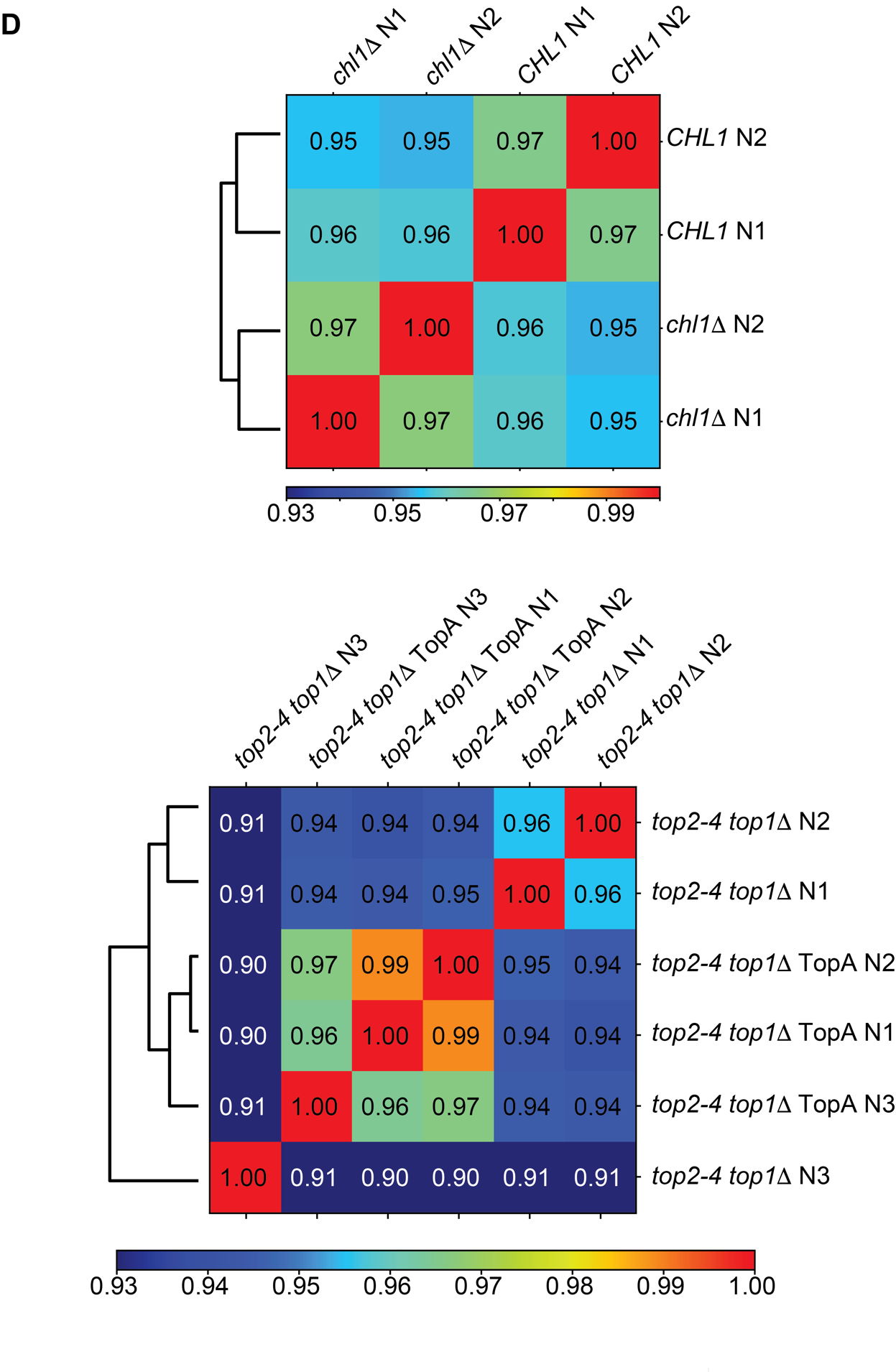
**

**Appendix Figure S3. Reproducibility between micro-C experiments.**

Spearman correlation plots were used to check data reproducibility between independent micro-C repeat experiments, used to prepare the indicated Figures. Note that cohesin-dependent chromatin loops are a relatively small feature in the context of a genome-wide chromatin interaction map. While repeat experiments typically show very close correlation, substantial similarities also exist between maps obtained from different genetic strain backgrounds and experimental conditions. The correlation plots relate to the experiments shown in (**A**) Figure 1, (**B**) Figure 2, (**C**) Figure 3, (**D**) Figure 4, and (**E**) Figure EV3.
