# Supplementary figures and images for: An extrinsic motor directs chromatin loop formation by cohesin
Source: EMBO J. 2024 Aug 19;43(19):3. doi: 10.1038/s44318-024-00202-5 (PMC11445435; doi:10.1038/s44318-024-00202-5)

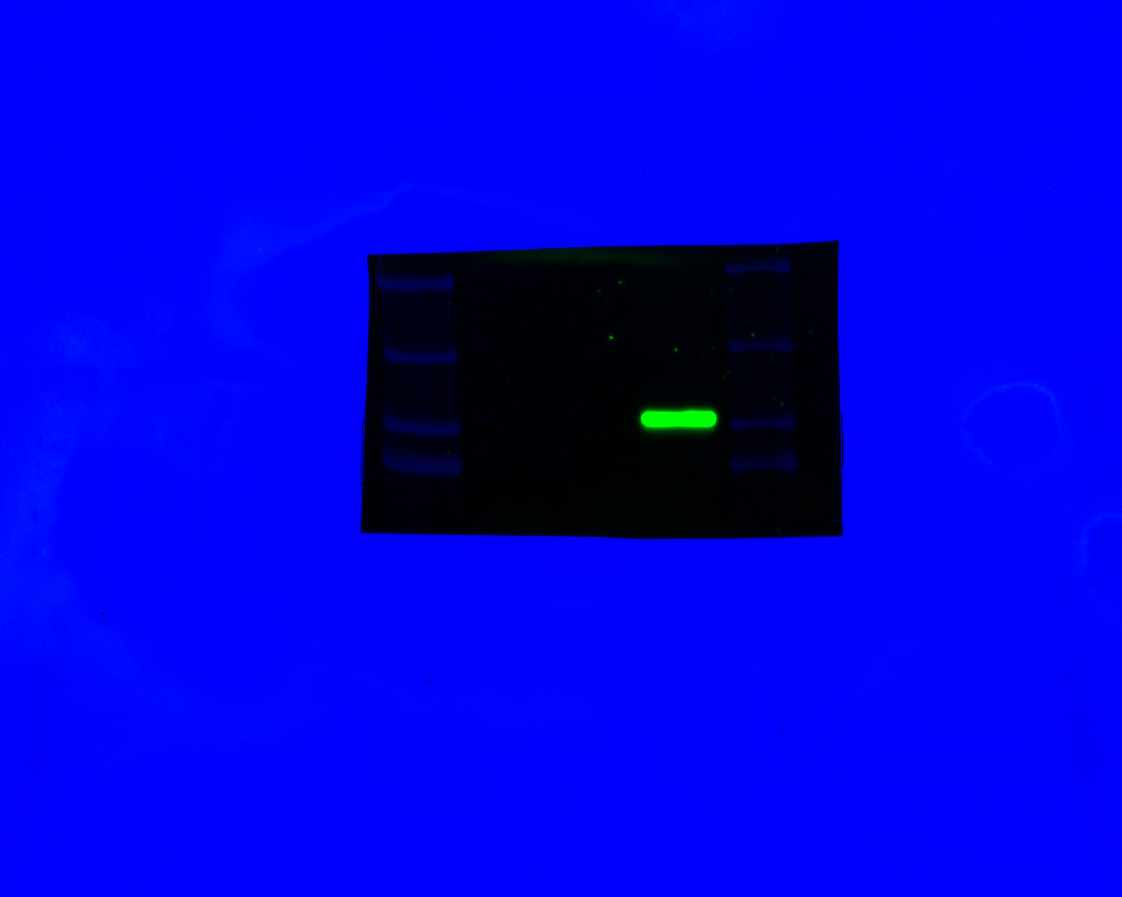

Supplement: Supplementary file 5 — Source data Fig. 4 [file 44318_2024_202_MOESM5_ESM.zip › Figure 4/Figure 4C/Merged_TopA_Ladder.tif]

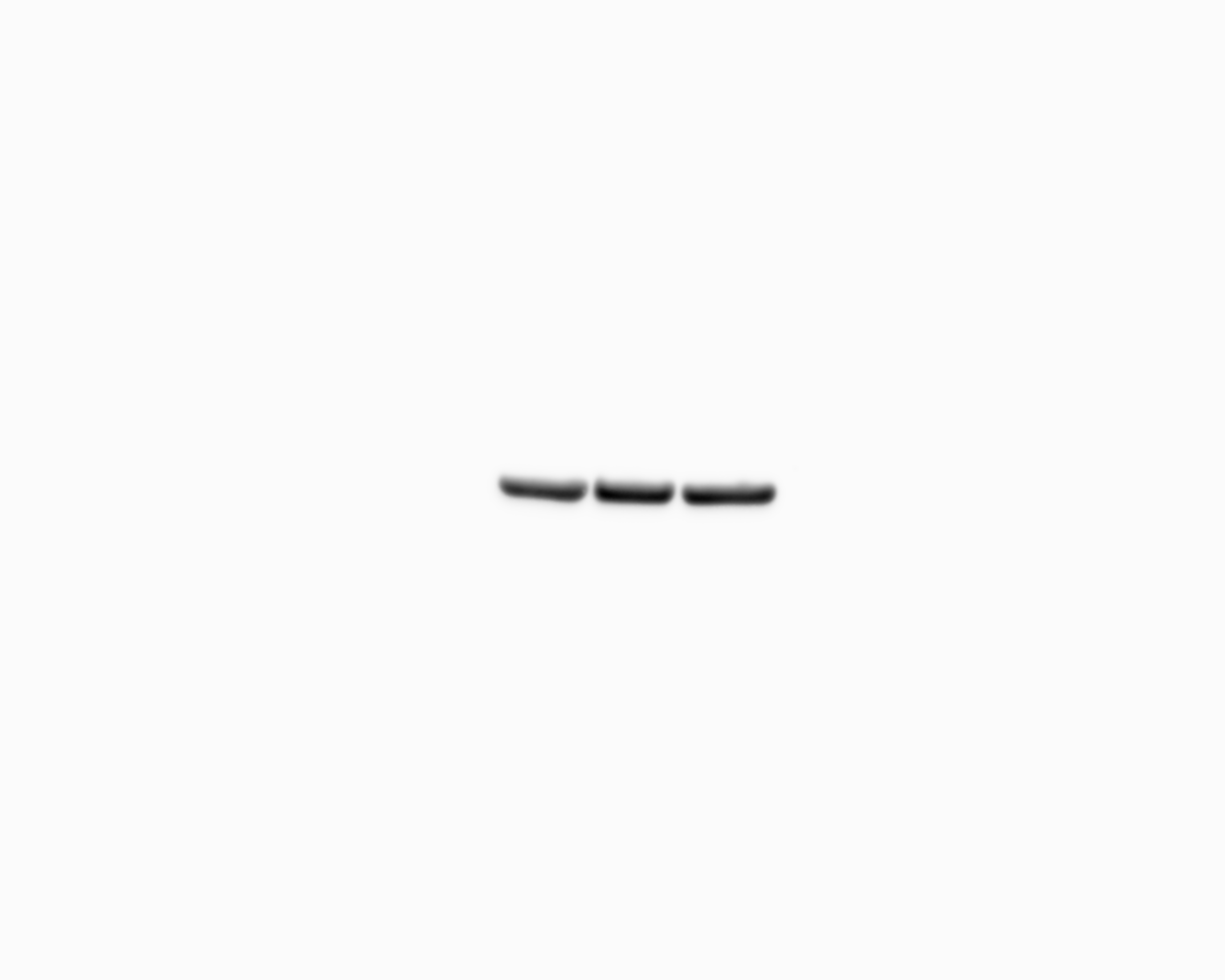

Supplement: Supplementary file 5 — Source data Fig. 4 [file 44318_2024_202_MOESM5_ESM.zip › Figure 4/Figure 4C/GAPDH.tif]

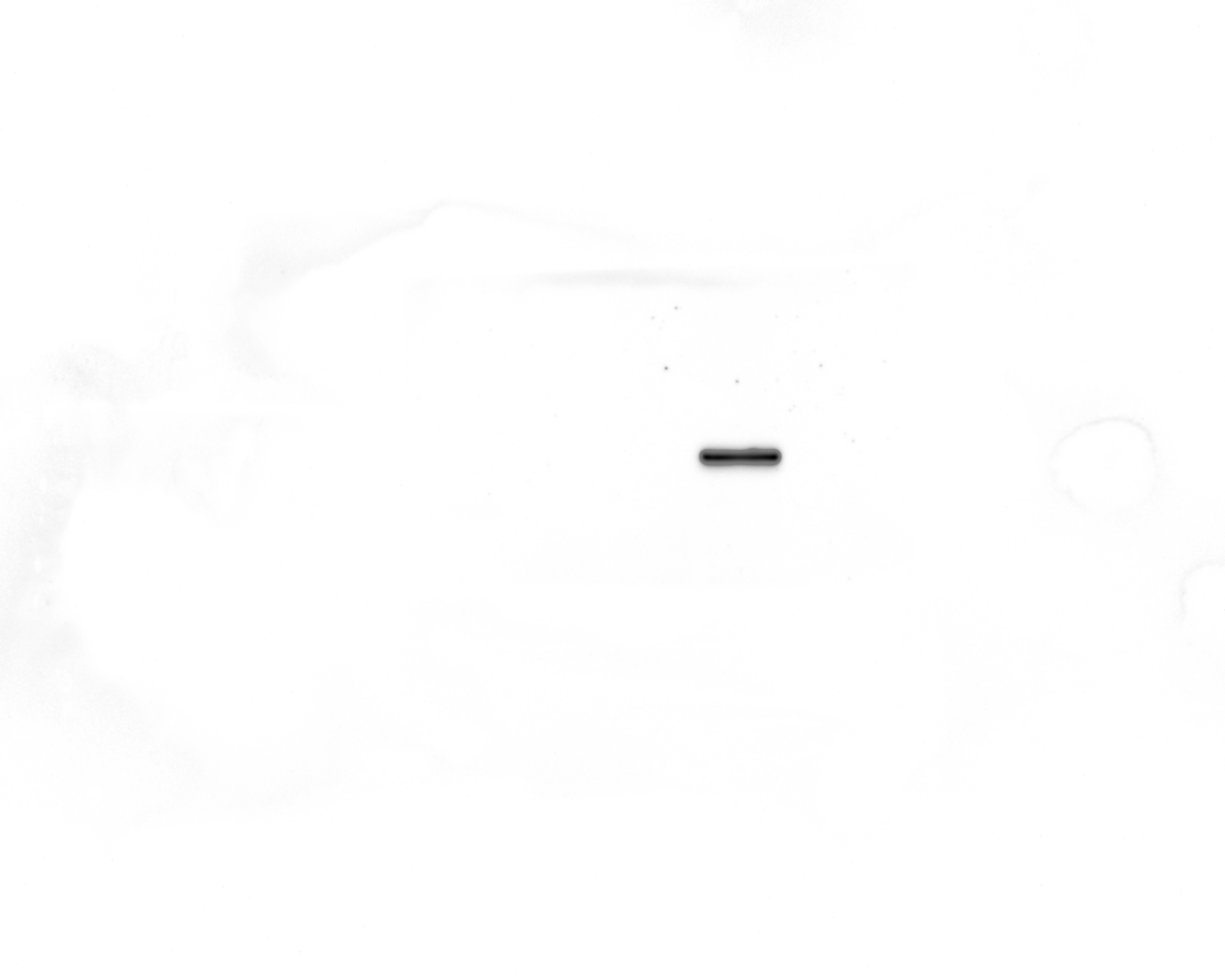

Supplement: Supplementary file 5 — Source data Fig. 4 [file 44318_2024_202_MOESM5_ESM.zip › Figure 4/Figure 4C/TopA.tif]

top1Δ  
top2-4

kDA

wt

+topA

250

150

100

75

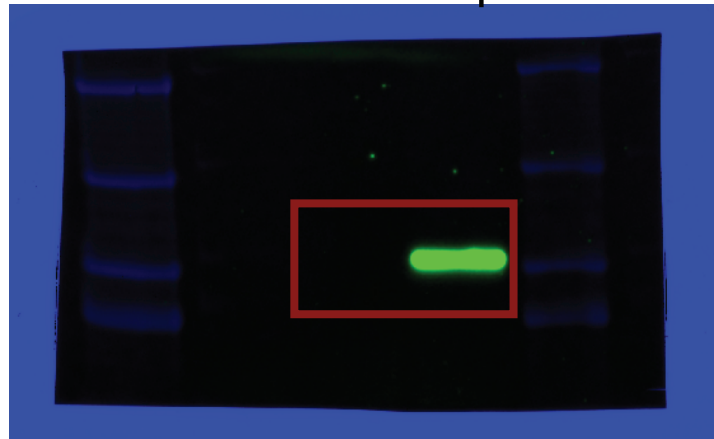

50

37

25

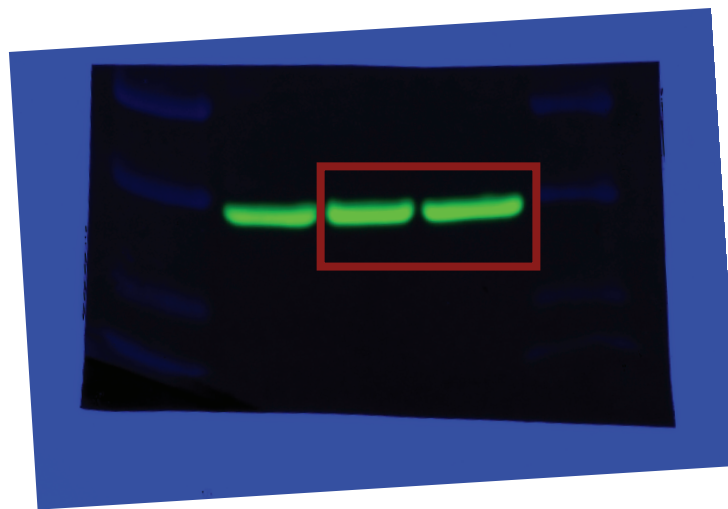

Supplement: Supplementary file 5 — Source data Fig. 4 [file 44318_2024_202_MOESM5_ESM.zip › Figure 4/Figure 4C/TopA GAPDH crop regions.pdf]

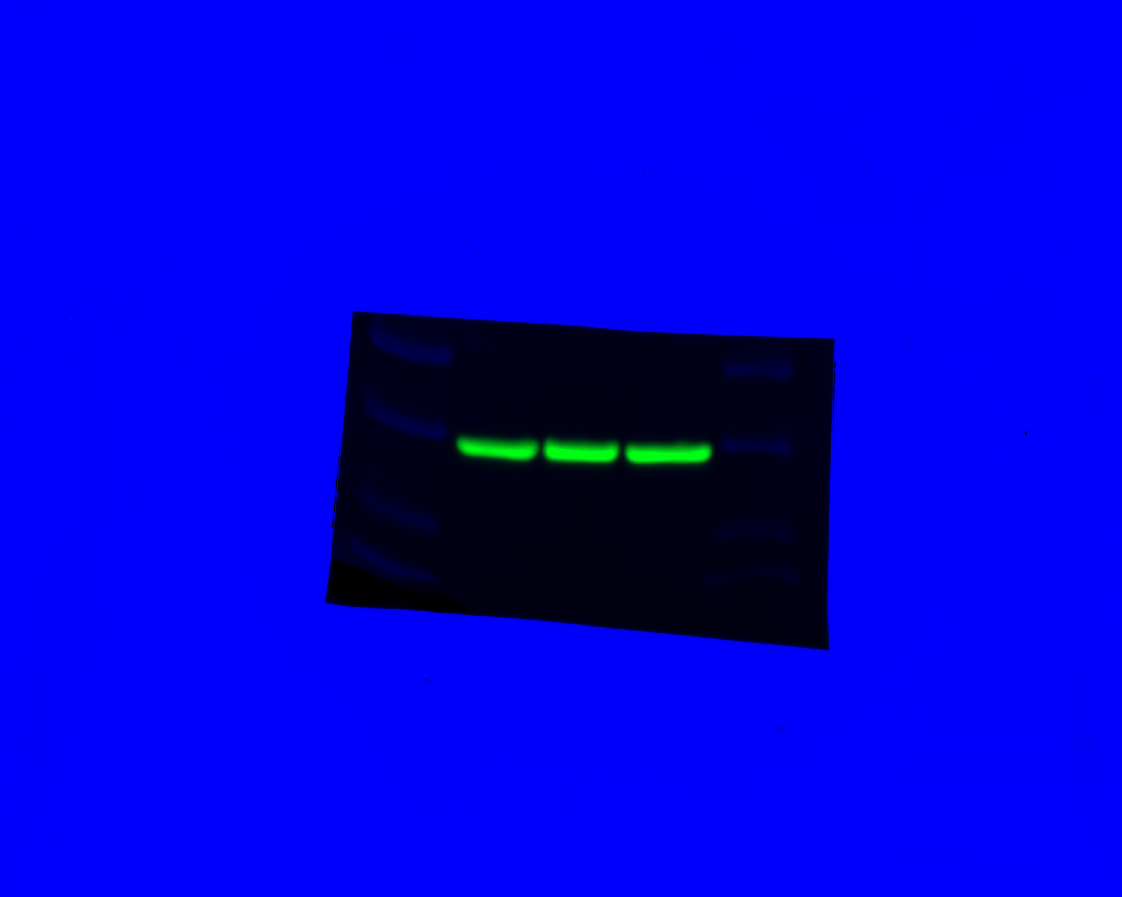

Supplement: Supplementary file 5 — Source data Fig. 4 [file 44318_2024_202_MOESM5_ESM.zip › Figure 4/Figure 4C/Merge_GAPDH_Ladder.tif]
