# Supplementary material for: An extrinsic motor directs chromatin loop formation by cohesin
Source: EMBO J. 2024 Aug 19;43(19):3. doi: 10.1038/s44318-024-00202-5 (PMC11445435; doi:10.1038/s44318-024-00202-5)
Supplement: Supplementary file 6 — Expanded View Figures [file 44318_2024_202_MOESM6_ESM.pdf]

Expanded View Figures

Figure EV1. Characterisation of $Scc3^{3E}$ and $Smc1^{4E}$ loop extrusion defective cohesin complexes.

(**A**) Purified wild type (wt), $Scc3^{3E}$-, and $Smc1^{4E}$-cohesin and cohesin loader were analysed by SDS-PAGE followed by Coomassie Blue staining. (**B**) Loop extrusion rates, measured as described (Higashi et al, 2021), of wt and $Smc1^{4E}$-cohesin, in the presence of loader and ATP ($n_{wt} = 37$, $n_{Smc1}{}^{4E} = 16$). Dashed and dotted lines represent the median and quartile ranges, respectively. Processive extrusion by $Smc1^{4E}$-cohesin suggests that this variant is defective in loop initiation but less so loop extension. Indeed, $Smc1^{4E}$-cohesin shows a greater median extrusion rate, which might arise if the small number of loop extrusion events by this variant are biased towards DNAs under low tension on which extrusion proceeds relatively faster. (**C**) DNA affinity of wt, $Scc3^{3E}$- and $Smc1^{4E}$-cohesin as measured by an electrophoretic mobility shift assay. Increasing cohesin concentrations were between 32 and 525 nM in 2-fold steps. (**D**) Assay to measure topological (high-salt-resistant) loading of wt, $Scc3^{3E}$- and $Smc1^{4E}$-cohesin onto DNA (Minamino et al, 2018), in the presence of the indicated components. An example agarose gel of the recovered DNA is shown, as well as quantification of the individual results from two independent repeat experiments. Bars show the means. (**E**) Loop extrusion assay as in Fig. 1C, but the flow cell was incubated with wt, $Scc3^{3E}$- or $Smc1^{4E}$-cohesin, loader and ATP in the absence of flow, before flow was applied to visualise loops. The fractions of DNA with loops were counted in three independent repeat experiments. Individual data points are shown, bars represent the mean and error bars the standard deviation ($n_{wt} = 224$, $n_{Scc3}{}^{3E} = 269$, $n_{Smc1}{}^{4E} = 633$). (**F**) As Fig. 1C in the presence of flow, but a buffer containing 100 mM NaCl was used. See the Methods for complete buffer descriptions. Bars represent the mean and error bars the standard deviation ($n_{wt} = 452$, $n_{Scc3}{}^{3E} = 295$, $n_{Smc1}{}^{4E} = 242$).

 © The Author(s)

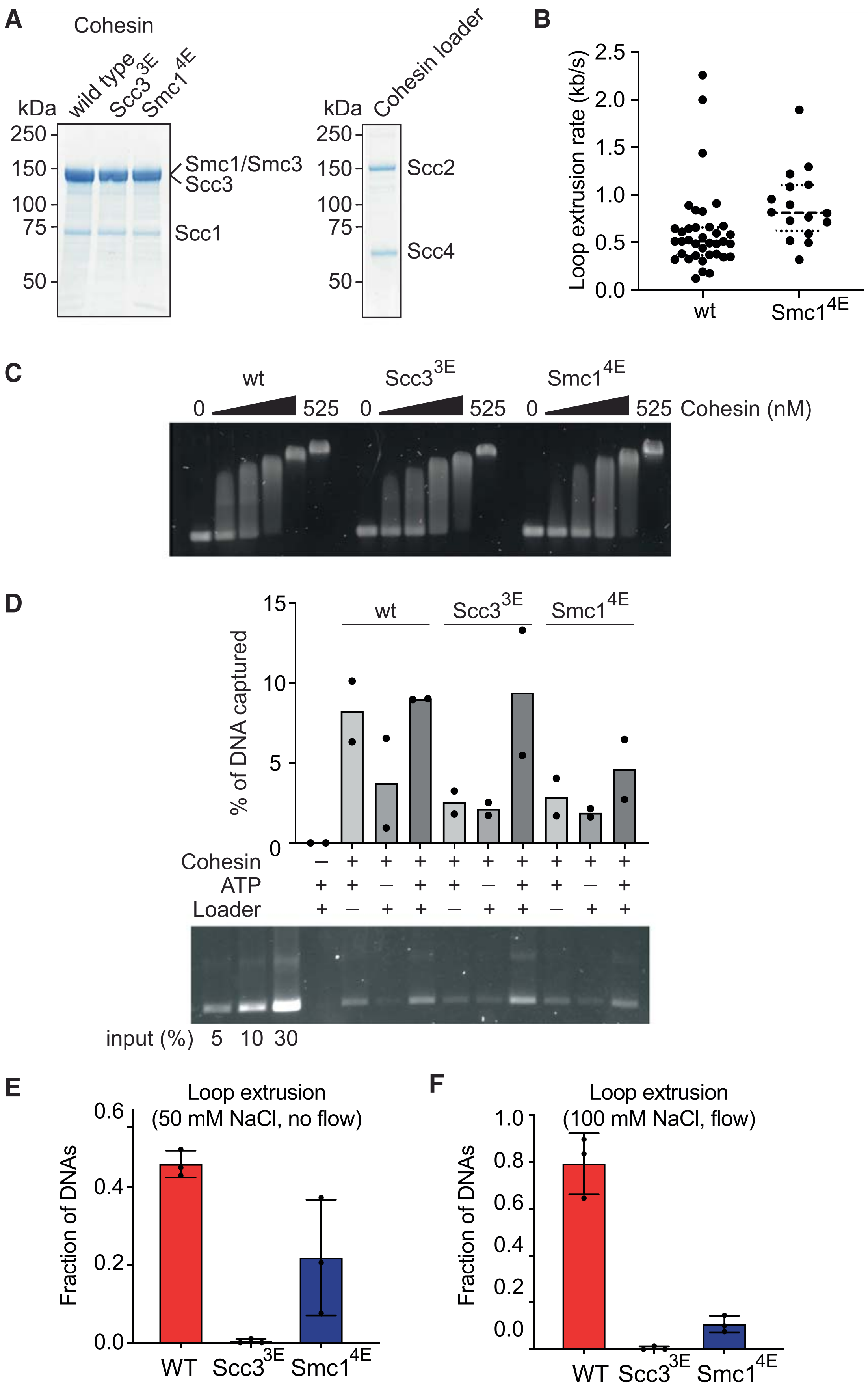
A
Cohesin
kDa
wild type
Scc3 3E
Smc1 4E
250
150
100
75
50
Smc1/Smc3
Scc3
Scc1
kDa
Cohesin loader
Scc2
Scc4
B
Loop extrusion rate (kb/s)
2.5
2.0
1.5
1.0
0.5
0.0
wt
Smc1 4E
C
wt
Scc3 3E
Smc1 4E
0
525
Cohesin (nM)
D
% of DNA captured
15
10
5
0
Cohesin
ATP
Loader
input (%)
5 10 30
E
Loop extrusion
(50 mM NaCl, no flow)
Fraction of DNAs
0.6
0.4
0.2
0.0
WT
Scc3 3E
Smc1 4E
F
Loop extrusion
(100 mM NaCl, flow)
Fraction of DNAs
1.0
0.8
0.6
0.4
0.2
0.0
WT
Scc3 3E
Smc1 4E

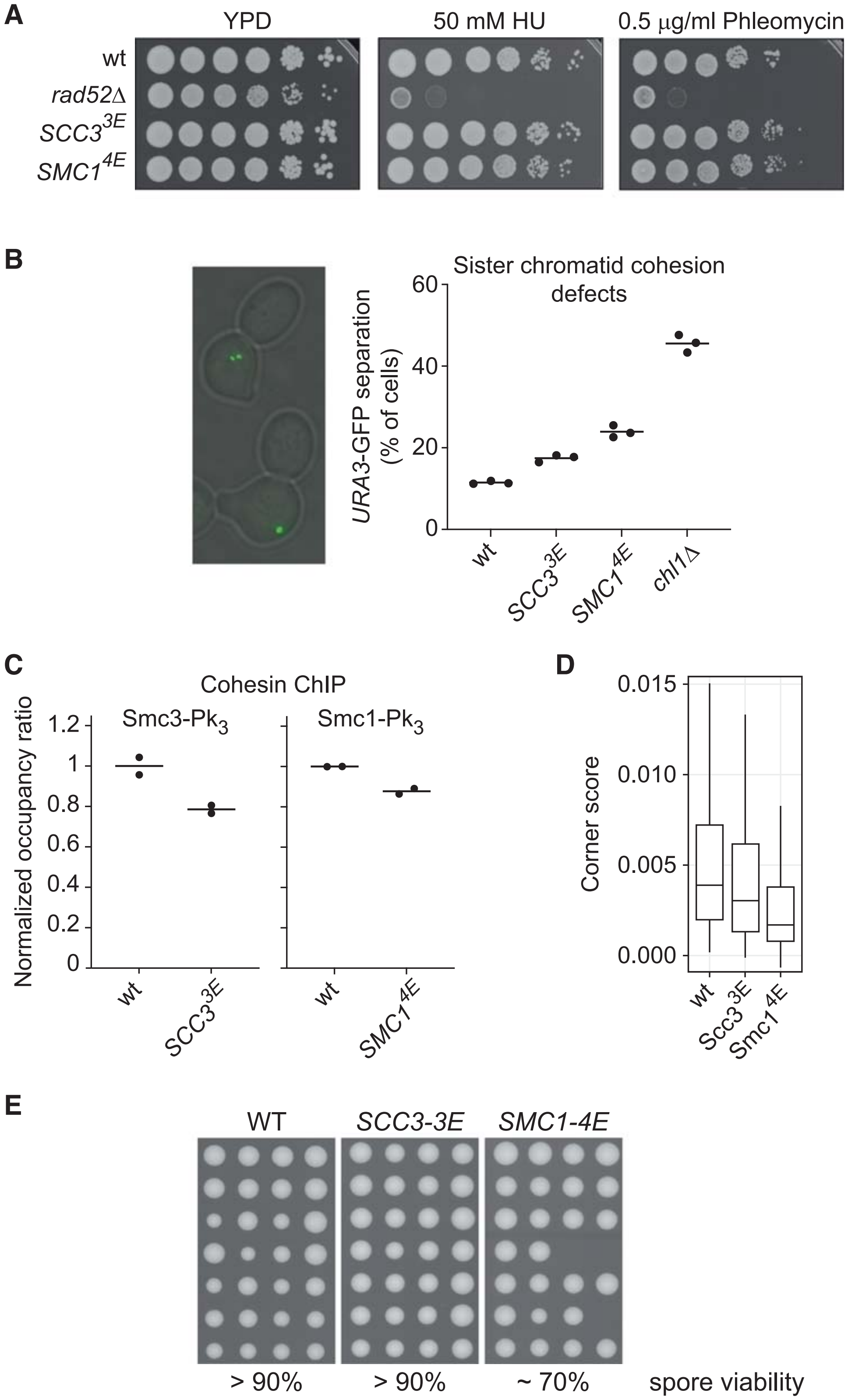
A
YPD
50 mM HU
0.5 μg/ml Phleomycin
wt
rad52Δ
SCC3^3E
SMC1^4E
B
Sister chromatid cohesion defects
URA3-GFP separation (% of cells)
60
40
20
0
wt
SCC3^3E
SMC1^4E
chl1Δ
C
Cohesin ChIP
Smc3-Pk3
Smc1-Pk3
Normalized occupancy ratio
1.2
1
0.8
0.6
0.4
0.2
0
wt
SCC3^3E
wt
SMC1^4E
D
Corner score
0.015
0.010
0.005
0.000
wt
Scc3^3E
Smc1^4E
E
WT
SCC3-3E
SMC1-4E
> 90%
> 90%
~ 70%
spore viability

 © The Author(s)

Figure EV2. Life without loop extrusion.

(**A**) 10-fold serial dilutions of cultures of the indicated genotypes were plated onto YPD agar plates containing the indicated compounds and grown at 30 °C for 2 days. A wt and a DNA repair deficient (*rad52Δ*) strain were included as controls. (**B**) Sister chromatid cohesion in G2/M arrested cells was monitored at the GFP-marked *URA3* locus (Michaelis et al, 1997). A representative image of two G2/M arrested cells with intact (left) or defective (right) sister chromatid cohesion is shown. The percentage of cells ($n = 100$) with two separated GFP dots were recorded in three independent repeat experiments. The means are represented by horizontal bars. A wild type (wt) and a cohesion establishment defective (*chl1Δ*; Samora et al, 2016) strain served as controls. (**C**) Overall cohesin ChIP enrichment ratios of wt, compared to Scc3^{3E}- and Smc1^{4E}-cohesin, relative to a *C. glabrata* spike-in. Cohesin ChIP used Smc3-Pk$_3$ in the Scc3^{3E} strain, or Smc1^{4E}-Pk$_3$, normalised against Smc3-Pk$_3$ and Smc1-Pk$_3$ wt control strains. (**D**) Corner score distributions of loops identified in the wild-type micro-C contact map and linked to cohesin anchors, sampled in the Scc3^{3E}- and Smc1^{4E}-maps ($n = 1060$). Box plots represent the median (centre), quartiles (box) and range (whiskers). (**E**) Tetrad dissection following sporulation of homozygous diploid wild type, *SCC3^{3E}* and *SMC1^{4E}* strains. Spore viability was calculated based on $n = 118/128$, 104/100 and 82/120 germinating and colony forming spores, respectively.

© The Author(s)

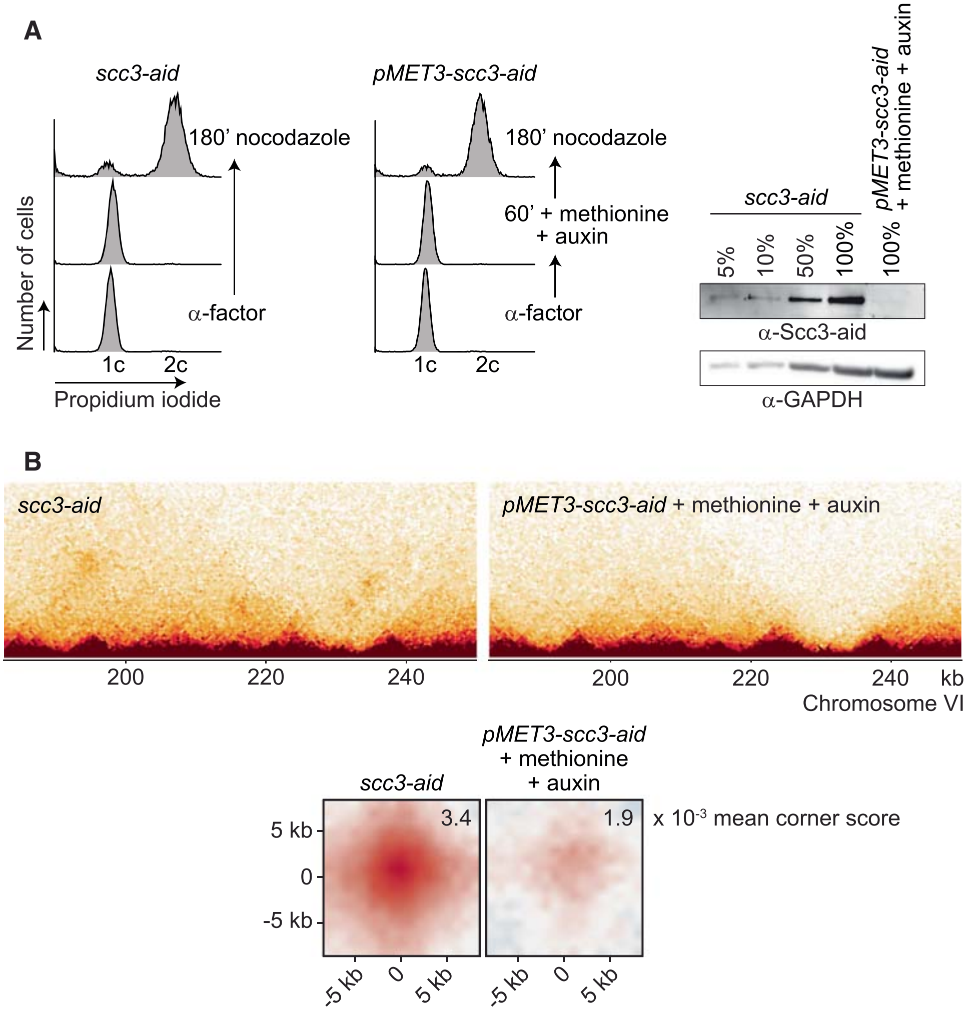

Figure EV3. Scc3 is required for chromatin loop formation.

(**A**) FACS analysis of DNA content, as well as experimental outline, of the experiment to deplete Scc3 by promoter shut-off and an auxin-inducible degron (*pMET-scc3-aid* cells). As a control, we used cells in which *scc3-aid* is expressed under control of its endogenous, methionine-insensitive promoter and to which we added methionine but not auxin during G1 arrest, before release into nocodazole-containing medium for arrest in G2/M. Scc3 depletion was confirmed by Western blotting. Serial dilutions of the control sample without auxin addition were loaded, as well as the depleted sample. Scc3 was detected using an α-aid-tag antibody (Cosmo Bio, CAC-APC004AM). GAPDH, detected by an α-GAPDH antibody (abcam, clone GA1R, ab125247) served as a loading control. (**B**) 500 bp-resolution merged micro-C contact maps from two independent experiments with Scc3-depleted *pMET-scc3-aid* and control *scc3-aid* cells. Aggregate chromatin loop profiles, detected by chromosight and linked to cohesin anchors in a wild-type strain without any cohesin alteration (Fig. 1C), were recorded in both present maps.

 © The Author(s)

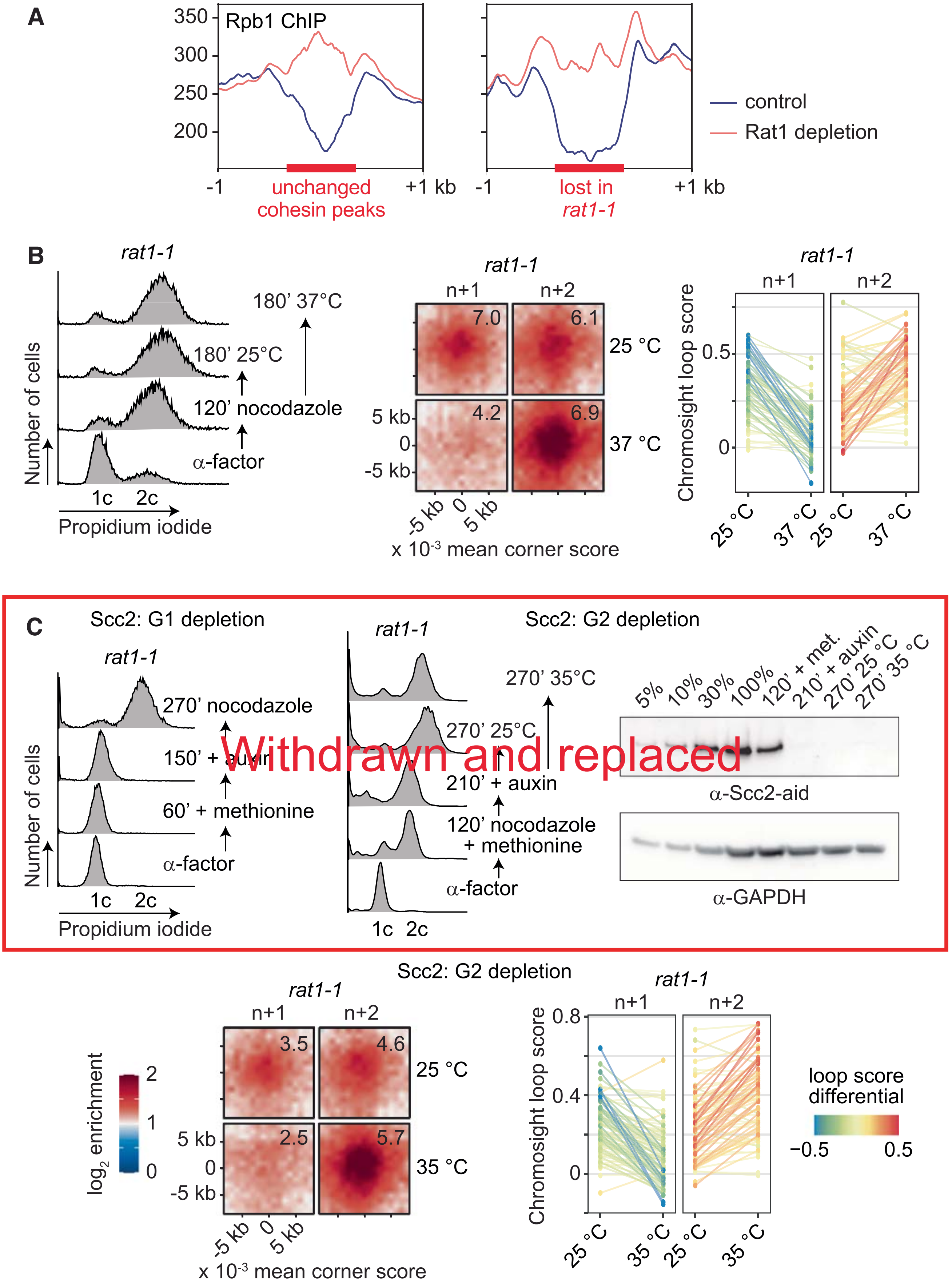
A
Rpb1 ChIP
350
300
250
200
-1
+1 kb
unchanged cohesin peaks
lost in rat1-1
control
Rat1 depletion
B
rat1-1
180' 37°C
180' 25°C
120' nocodazole
α-factor
Number of cells
1c 2c
Propidium iodide
n+1 n+2
7.0 6.1
4.2 6.9
25 °C
37 °C
5 kb
0
-5 kb
x 10-3 mean corner score
Chromosight loop score
0.5
0
25 °C 37 °C
C
Withdrawn and replaced
Scc2: G1 depletion
270' nocodazole
150' + auxin
60' + methionine
Scc2: G2 depletion
270' 35°C
270' 25°C
210' + auxin
120' nocodazole + methionine
5% 10% 30% 100% 120' + met. 210' + auxin 270' 25 °C 270' 35 °C
α-Scc2-aid
α-GAPDH
3.5 4.6
2.5 5.7
35 °C
log2 enrichment
2
1
0
0.8
0.4
loop score differential
−0.5 0.5

Figure EV4. Transcription expands cohesin-mediated chromatin loops.

(**A**) Characterisation of cohesin peaks that are displaced following *rat1-1* inactivation. Aggregated Rpb1 ChIP profiles (Baejen et al, 2017) are shown over scaled cohesin peak regions, and their surroundings, that remained either unchanged or that were lost following *rat1-1* inactivation. Before Rat1 depletion (anchor away was used by Baejen et al, 2017), cohesin peaks that will be displaced show strict Rpb1 avoidance. In contrast, cohesin peaks that will remain unchanged were already partly Rpb1 occupied. Following Rat1 depletion, Rpb1 broadly overlapped with both type of regions. We confirmed that differing cohesin peak widths did not cause these differences. To conduct these analyses, raw sequences from (Baejen et al, 2017) were aligned to the S288C genome for analysis using the standard nf core chipseq procedure. Bam files were then converted to BigWigs using bamCoverage with normalizeUsing RPKM and ignoreDuplicates parameters. Binsize was selected at 20 bp and data were smoothed over 3 bins. For comparison we overlaid our previous cohesin (Scc1) ChIP microarray analysis (Ocampo-Hafalla et al, 2016) and selected peaks exclusive to control cells. Peaks longer than 4000 bp or shorter than 500 bp were excluded from the analysis. (**B**) FACS analysis of DNA content of the cells in the experiment shown in Fig. 2C, together with an experimental outline. Aggregate chromatin profiles of loops ($n = 91$), identified as in Fig. 2B, and a graph depicting the *rat1-1* dependent loop score changes. (**C**) FACS analyses of DNA content of the cells in the experiment shown in Fig. 2D, together with experimental outlines. Western blot analysis confirmed Scc2 depletion by an auxin-inducible degron. Samples at the indicated times in the experiment are shown. Scc2 was detected using the aid-tag antibody. Tubulin served as a loading control. Aggregate loop profiles ($n = 52$) and a graph depicting the *rat1-1* dependent loop score changes are shown.

© The Author(s)

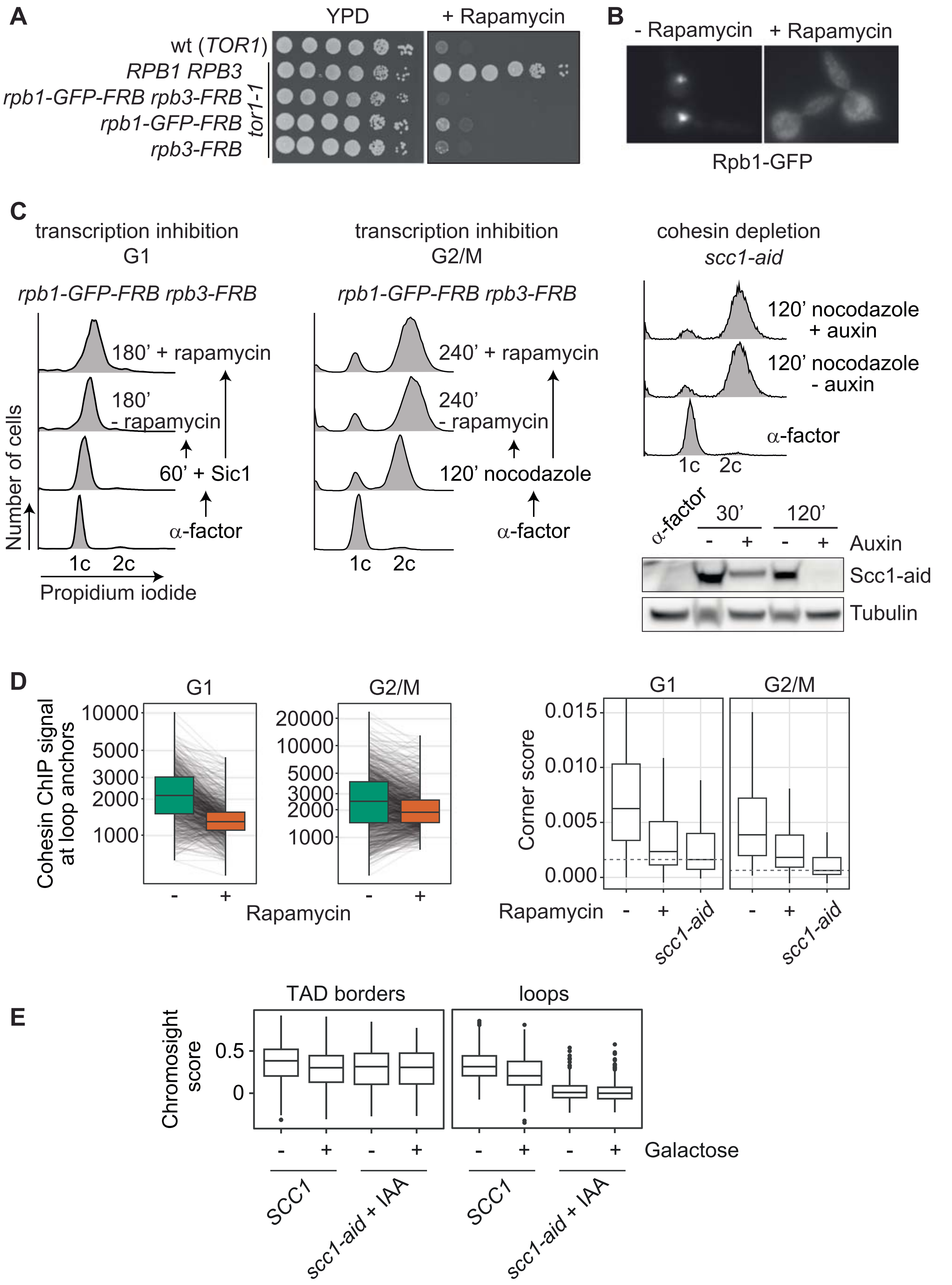
A
YPD
+ Rapamycin
wt (TOR1)
RPB1 RPB3
rpb1-GFP-FRB rpb3-FRB
rpb1-GFP-FRB
rpb3-FRB
tor1-1
B
- Rapamycin
+ Rapamycin
Rpb1-GFP
C
transcription inhibition G1
rpb1-GFP-FRB rpb3-FRB
180' + rapamycin
180' - rapamycin
60' + Sic1
α-factor
Number of cells
1c 2c
Propidium iodide
transcription inhibition G2/M
rpb1-GFP-FRB rpb3-FRB
240' + rapamycin
240' - rapamycin
120' nocodazole
α-factor
1c 2c
cohesin depletion
scc1-aid
120' nocodazole + auxin
120' nocodazole - auxin
α-factor
1c 2c
α-factor
30'
120'
Auxin
Scc1-aid
Tubulin
D
Cohesin ChIP signal at loop anchors
G1
G2/M
Rapamycin
Corner score
scc1-aid
E
TAD borders
loops
Chromosight score
Galactose
SCC1
scc1-aid + IAA

© The Author(s)

Figure EV5. Transcription inhibition and its effect on cohesin-mediated chromatin loops.

(**A**) 10-fold serial dilutions of cultures of the indicated genotypes were plated onto YPD agar plates, with or without 2 µg/ml added rapamycin, and grown at 30 °C for 2 days. A strain in which both Rpb1 and Rpb3 subunits of RNA polymerase II were fused to FRB showed a tighter response to rapamycin, as compared to strains with either one of the fusions. (**B**) An example of Rpb1-GFP-FRB relocation from the nucleus to the cytoplasm after one hour 2 µg/ml rapamycin treatment. Cells show the typical elongated bud shape of Sic1-induced late G1 arrest (Lopez-Serra et al, 2013). (**C**) FACS analysis of DNA content of the cells in the experiment shown in Fig. 3, as well as an experimental outline. Western blot analysis confirmed Scc1-aid depletion by its auxin-inducible degron, at 30 min and 120 min (the time of cell harvest) after release from α-factor synchronisation. Scc1 was detected with the α-aid antibody, tubulin served as the loading control and was detected with a mouse monoclonal α-Tub1 antibody (clone TAT-1). (**D**) Cohesin ChIP signal intensity distributions at loop anchors (normalised mean reads), in the absence or presence of rapamycin, in both the G1 and G2/M synchronised cultures. Grey lines connect individual ChIP signal intensities under the two conditions (G1: $n = 1059$, G2: $n = 1447$). Corner score distributions of the corresponding loops (G1: $n = 788$, G2: $n = 1060$), before and after transcription inhibition, as well as of the same loop positions sampled following Scc1 depletion, are shown alongside. Box plots represent the median (centre), quartiles (box) and range (whiskers). Baseline corner scores in the absence of cohesin are indicated by dashed lines. (**E**) Chromosight TAD boundary ($n = 822$) and Chromosight loop score ($n = 1300$) distributions, detected in *SCC1* cells grown in glucose (Fig. 5) and recorded from the maps under the indicated experimental conditions. Box plots represent the median (centre), quartiles (box) and range (whiskers).

© The Author(s)
