# Supplementary figures and images for: Author Correction: An extrinsic motor directs chromatin loop formation by cohesin
Source: EMBO J. 2025 Jan 31;44(5):1563–8. doi: 10.1038/s44318-024-00341-9 (PMC11876300; doi:10.1038/s44318-024-00341-9)

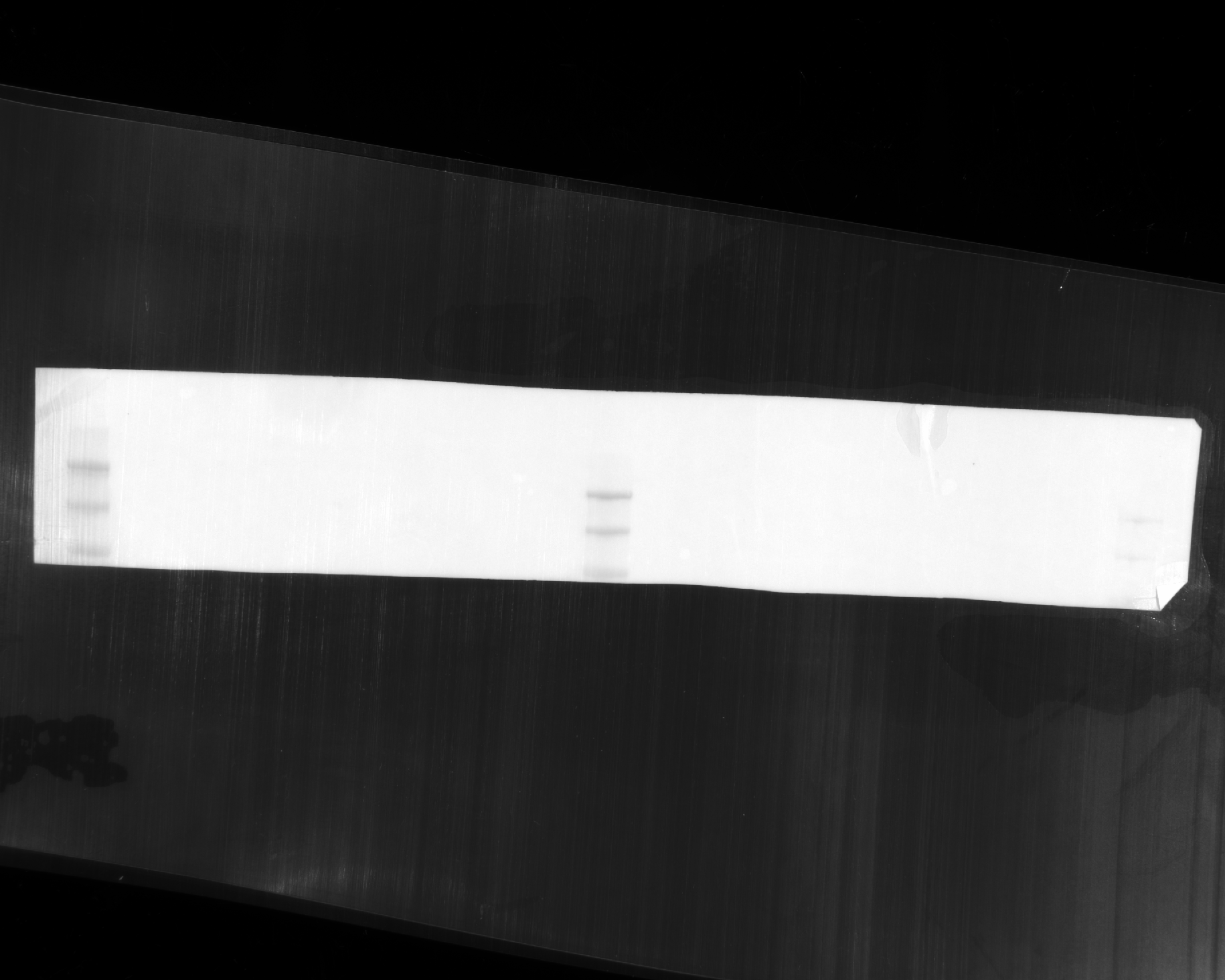

Supplement: Supplementary file 1 — Source Data EV4C_Corrected [file 44318_2024_341_MOESM1_ESM.zip › EV4C_Corrected_WB_Raw_Data/EV4C_Scc2-aid/Networked ChemiDoc 2024-09-19 15h44m21s(Colorimetric).tif]

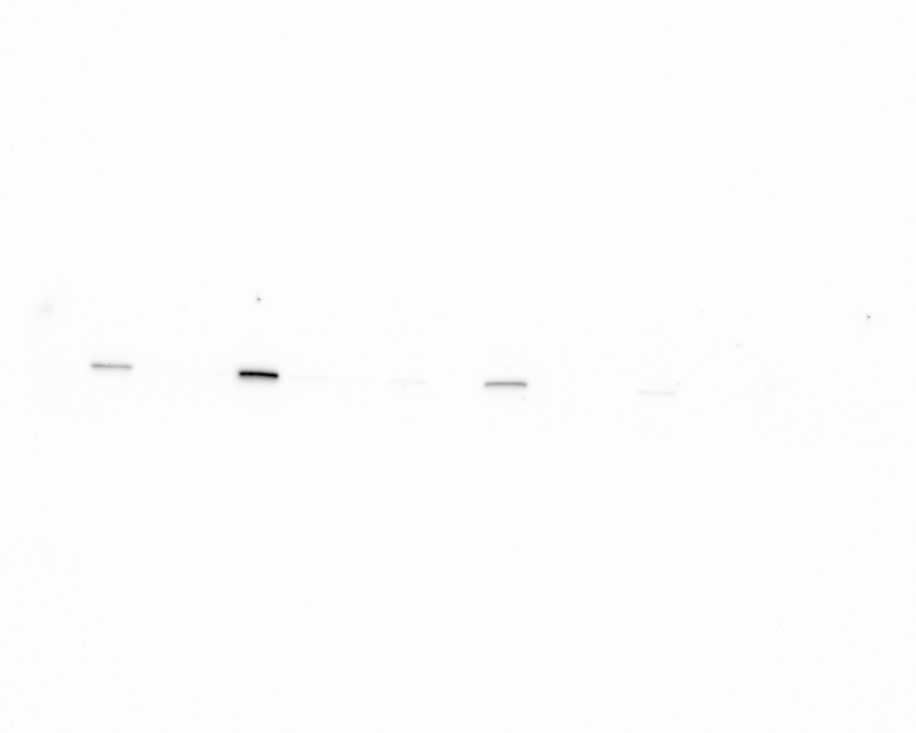

Supplement: Supplementary file 1 — Source Data EV4C_Corrected [file 44318_2024_341_MOESM1_ESM.zip › EV4C_Corrected_WB_Raw_Data/EV4C_Scc2-aid/Networked ChemiDoc 2024-09-19 15h05m38s(Chemiluminescence).raw16.png]

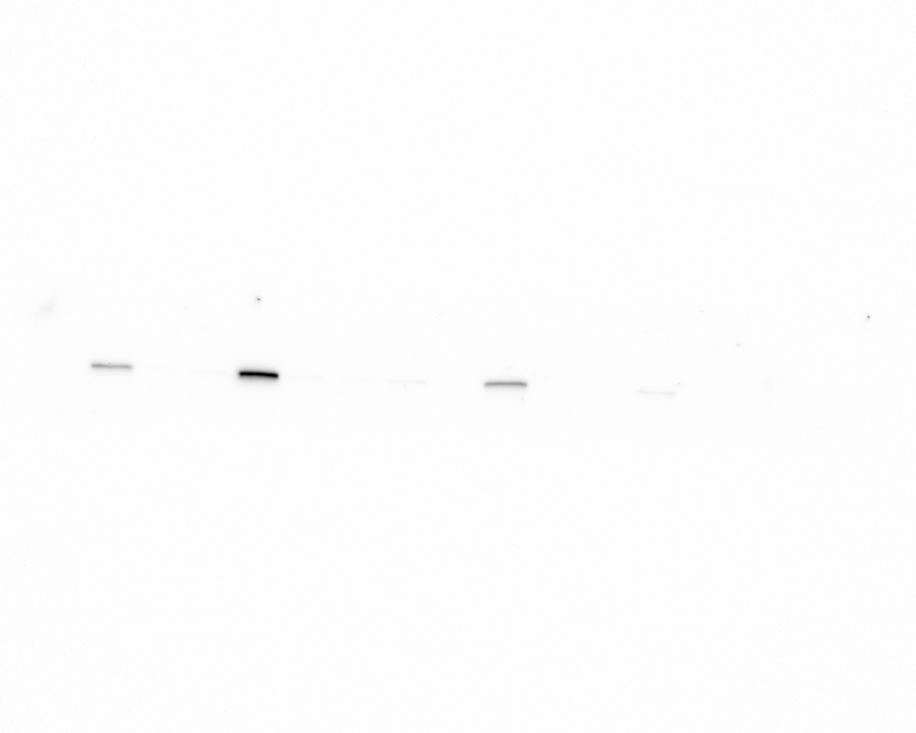

Supplement: Supplementary file 1 — Source Data EV4C_Corrected [file 44318_2024_341_MOESM1_ESM.zip › EV4C_Corrected_WB_Raw_Data/EV4C_Scc2-aid/Networked ChemiDoc 2024-09-19 15h05m38s(Chemiluminescence).raw16.tif]

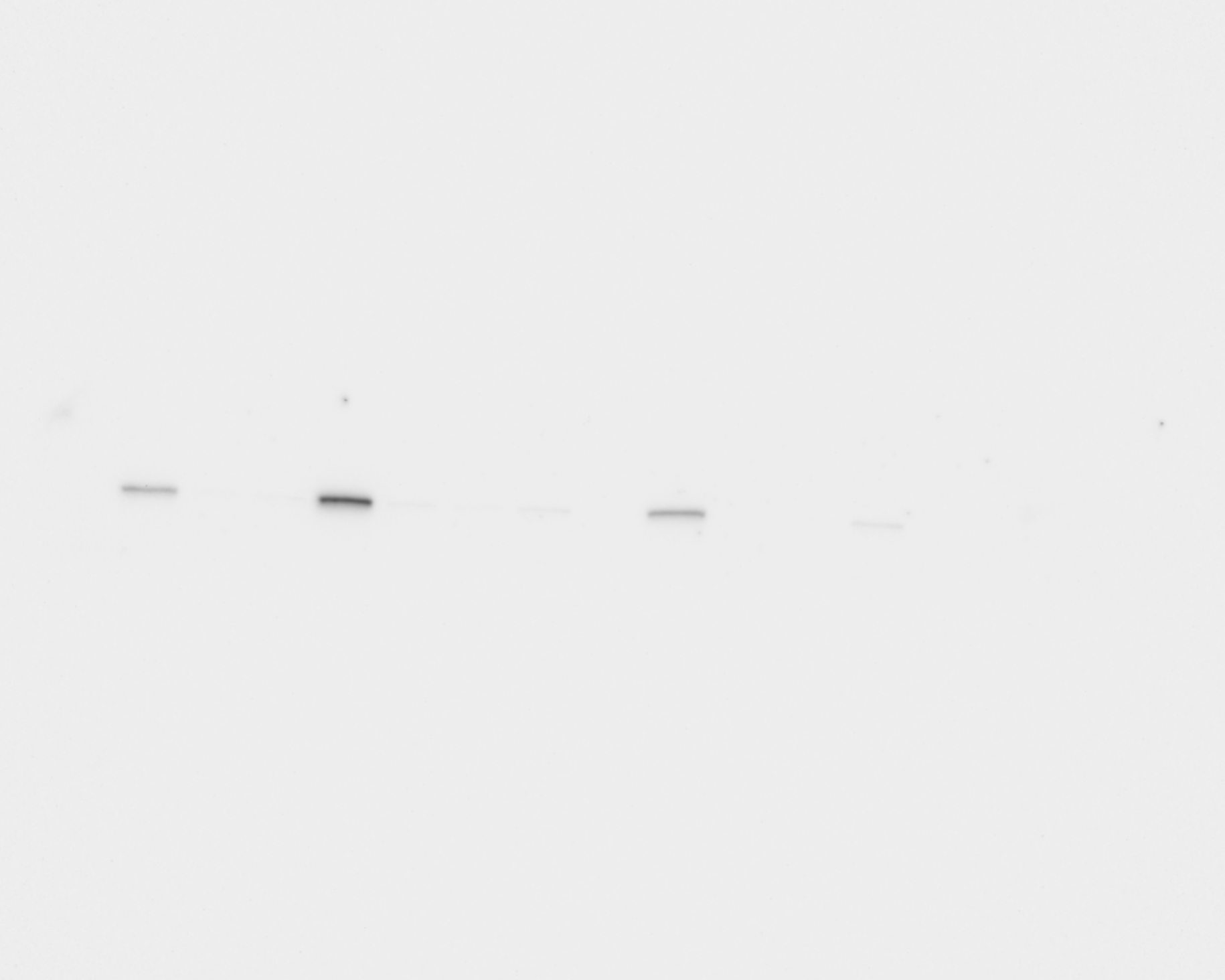

Supplement: Supplementary file 1 — Source Data EV4C_Corrected [file 44318_2024_341_MOESM1_ESM.zip › EV4C_Corrected_WB_Raw_Data/EV4C_Scc2-aid/Networked ChemiDoc 2024-09-19 15h05m38s(Chemiluminescence).tif]

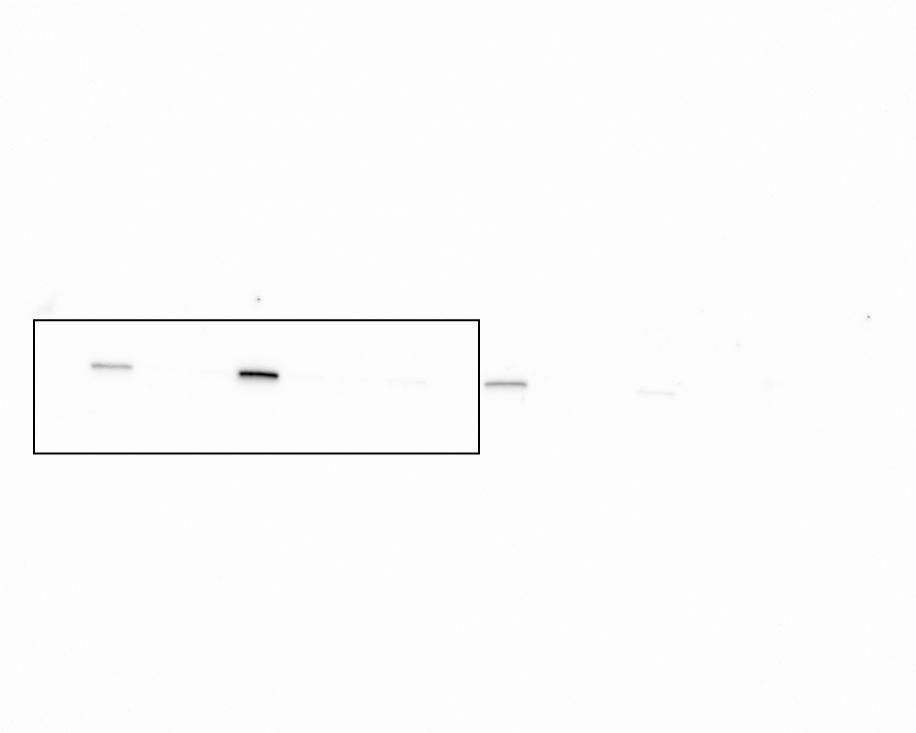

Supplement: Supplementary file 1 — Source Data EV4C_Corrected [file 44318_2024_341_MOESM1_ESM.zip › EV4C_Corrected_WB_Raw_Data/EV4C_Scc2-aid/Networked ChemiDoc 2024-09-19 15h05m38s(Chemiluminescence).raw16_Annotated.png]

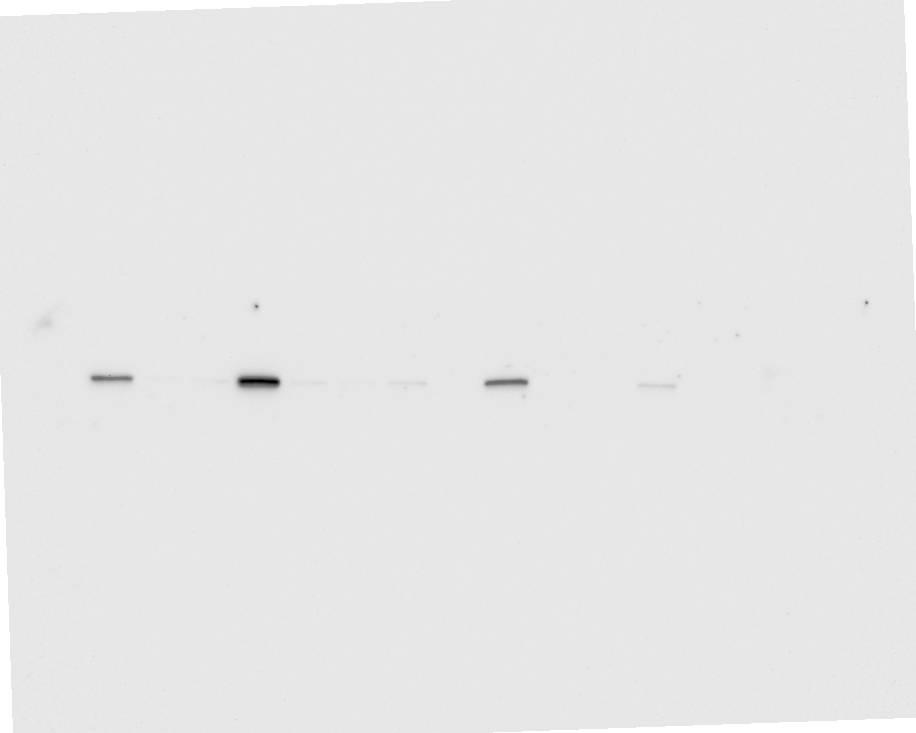

Supplement: Supplementary file 1 — Source Data EV4C_Corrected [file 44318_2024_341_MOESM1_ESM.zip › EV4C_Corrected_WB_Raw_Data/EV4C_Scc2-aid/Networked ChemiDoc 2024-09-19 15h05m38s(Chemiluminescence)ROTATE.raw16.tif]

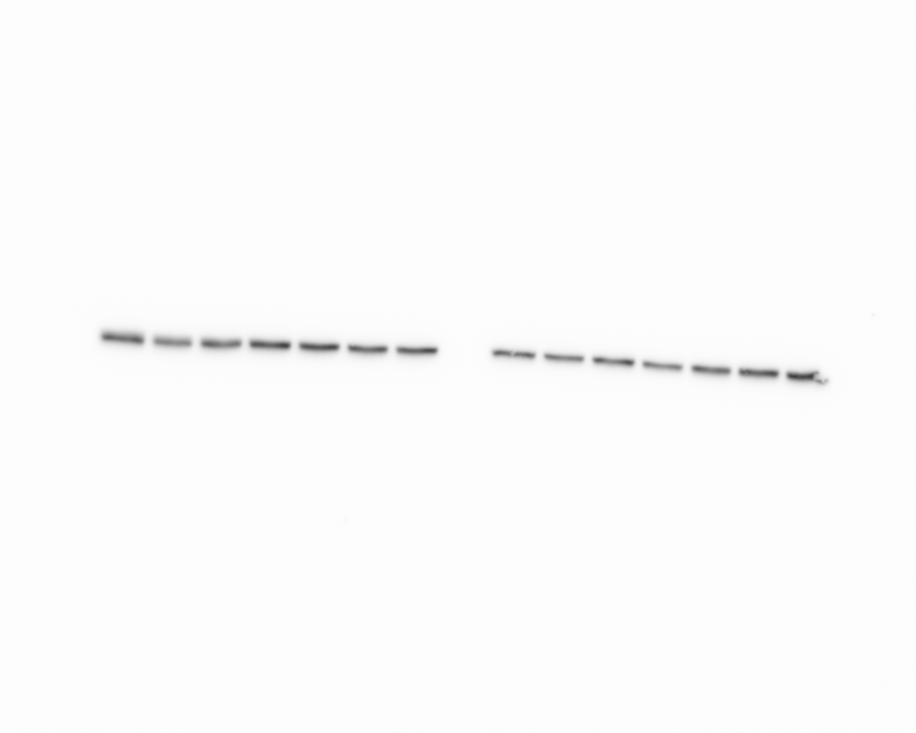

Supplement: Supplementary file 1 — Source Data EV4C_Corrected [file 44318_2024_341_MOESM1_ESM.zip › EV4C_Corrected_WB_Raw_Data/EV4C_Tubulin/Networked ChemiDoc 2024-09-19 15h46m05s(Chemiluminescence).raw16.tif]

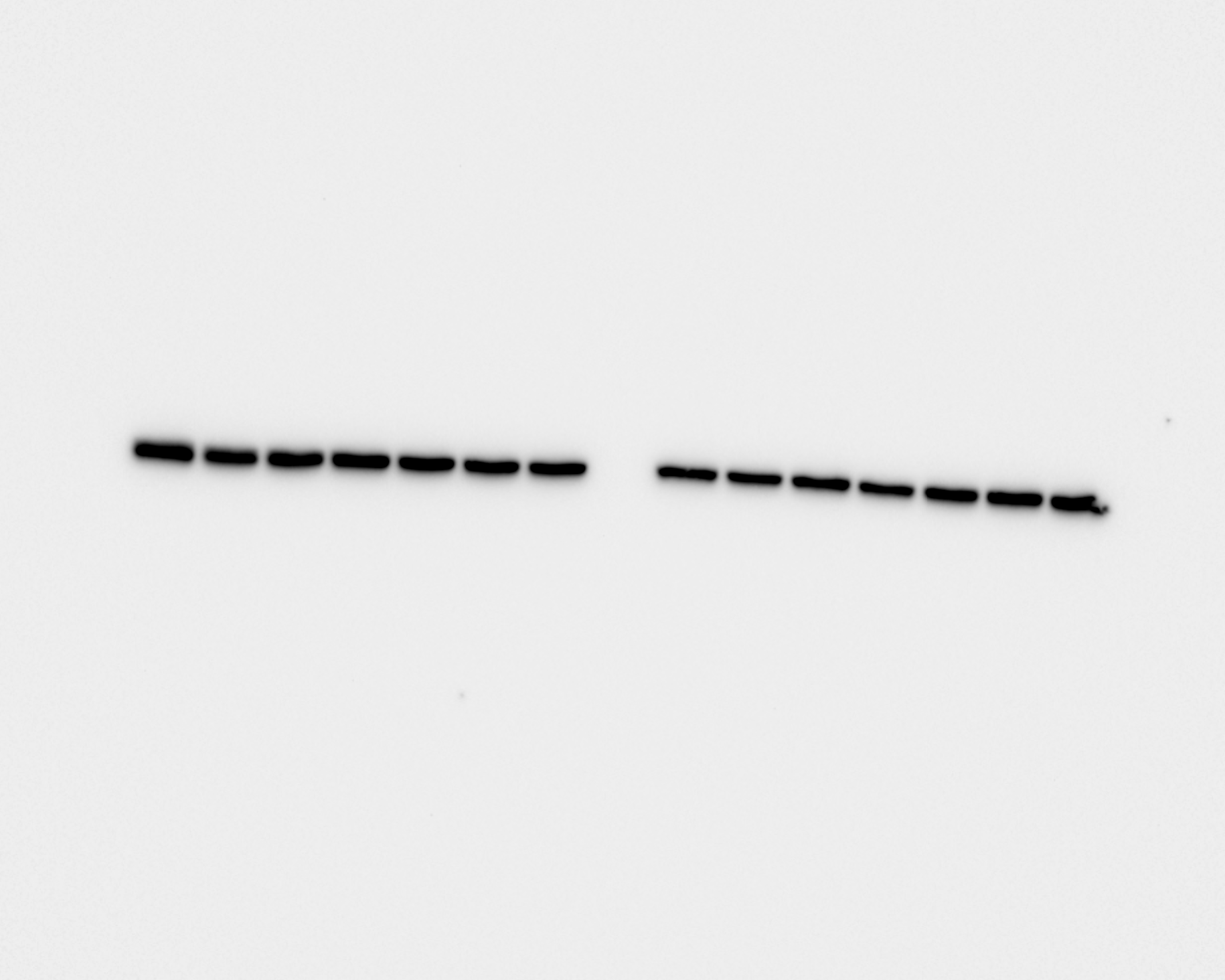

Supplement: Supplementary file 1 — Source Data EV4C_Corrected [file 44318_2024_341_MOESM1_ESM.zip › EV4C_Corrected_WB_Raw_Data/EV4C_Tubulin/Networked ChemiDoc 2024-09-19 15h46m05s(Chemiluminescence).tif]

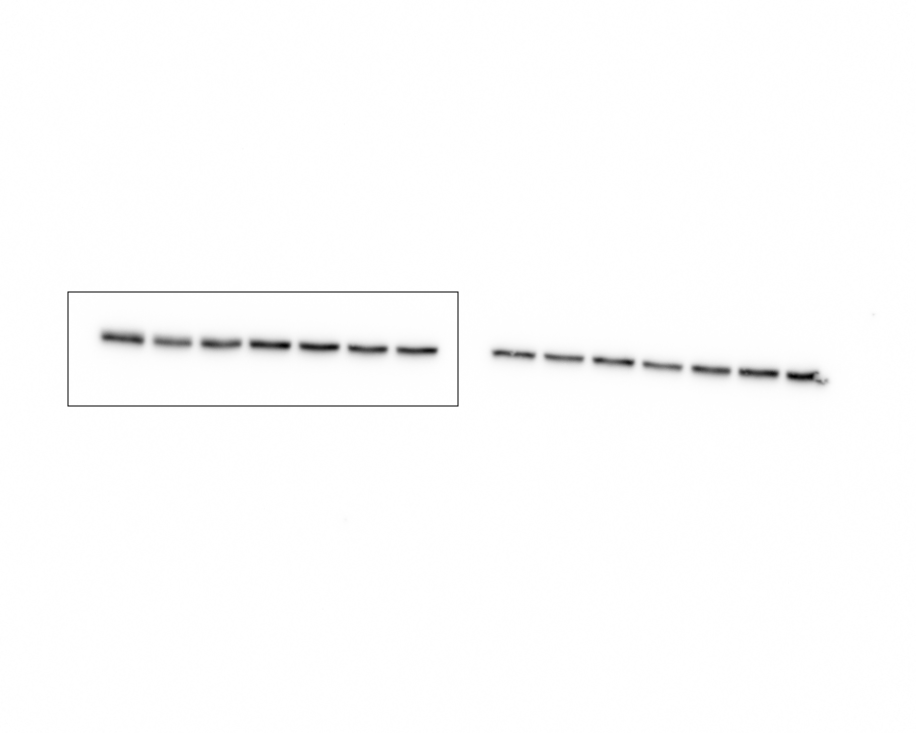

Supplement: Supplementary file 1 — Source Data EV4C_Corrected [file 44318_2024_341_MOESM1_ESM.zip › EV4C_Corrected_WB_Raw_Data/EV4C_Tubulin/Networked ChemiDoc 2024-09-19 15h46m05s(Chemiluminescence).raw16_Annotated.png]

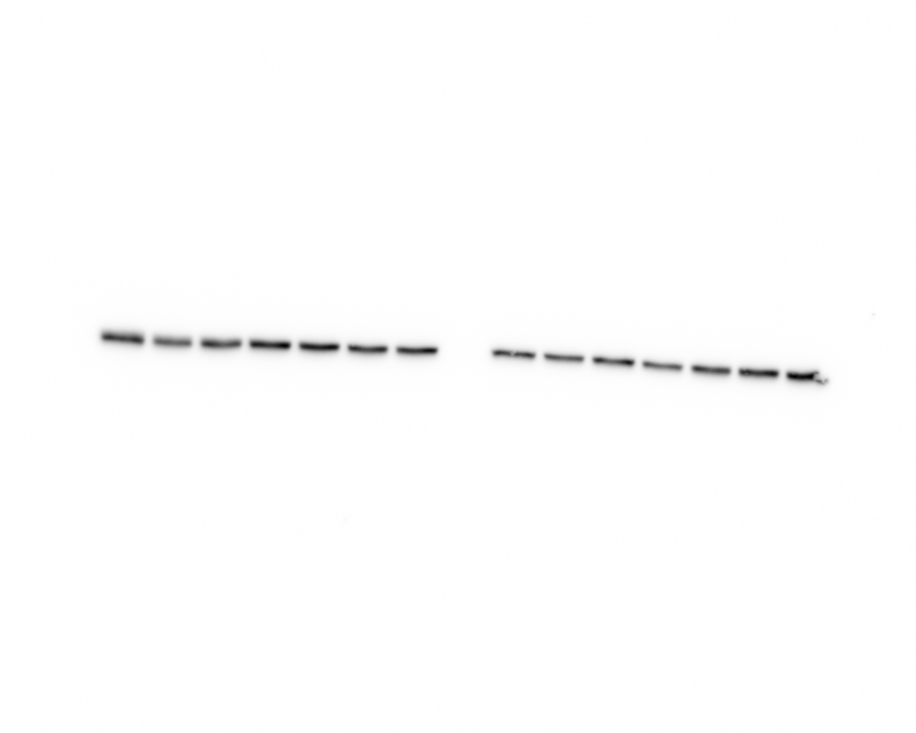

Supplement: Supplementary file 1 — Source Data EV4C_Corrected [file 44318_2024_341_MOESM1_ESM.zip › EV4C_Corrected_WB_Raw_Data/EV4C_Tubulin/Networked ChemiDoc 2024-09-19 15h46m05s(Chemiluminescence).raw16.png]

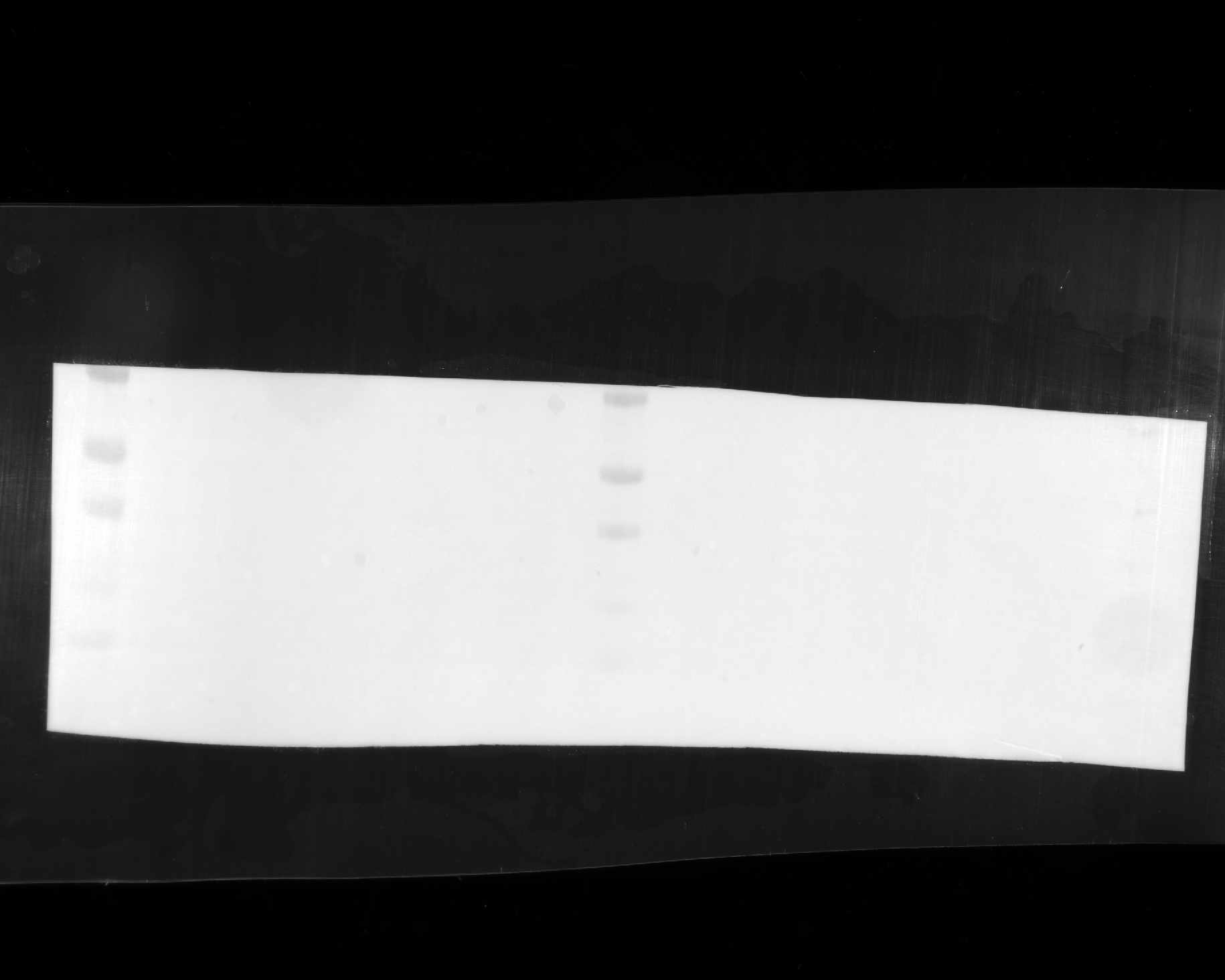

Supplement: Supplementary file 1 — Source Data EV4C_Corrected [file 44318_2024_341_MOESM1_ESM.zip › EV4C_Corrected_WB_Raw_Data/EV4C_Tubulin/Networked ChemiDoc 2024-09-19 15h45m07s(Colorimetric).tif]

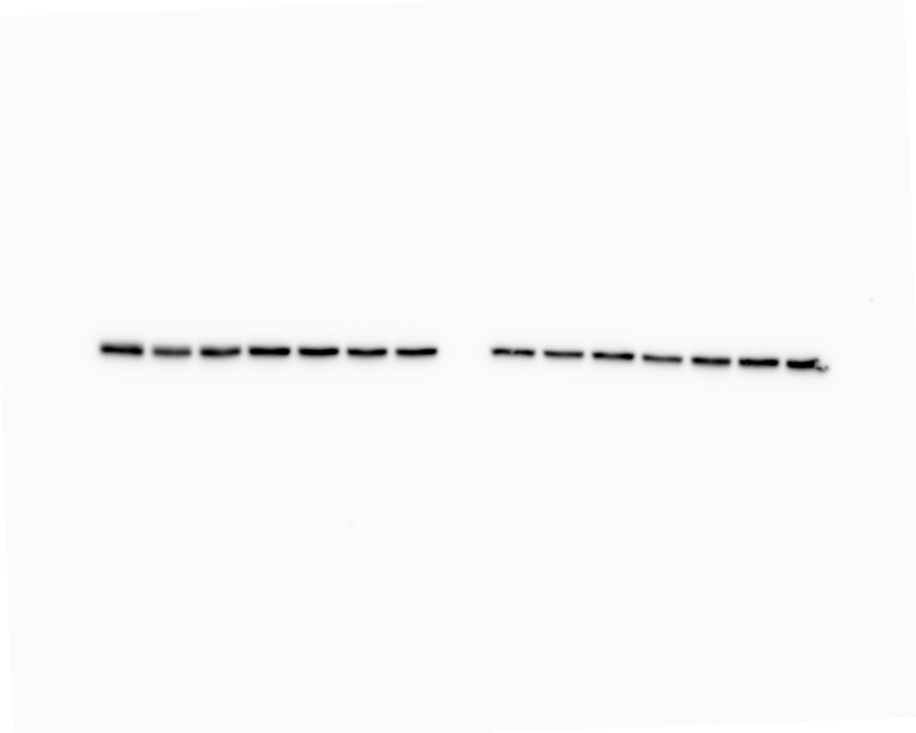

Supplement: Supplementary file 1 — Source Data EV4C_Corrected [file 44318_2024_341_MOESM1_ESM.zip › EV4C_Corrected_WB_Raw_Data/EV4C_Tubulin/Networked ChemiDoc 2024-09-19 15h46m05s(Chemiluminescence)ROTATE.raw16.tif]
